# Supplementary material for: Tufas indicate prolonged periods of water availability linked to human occupation in the southern Kalahari
Source: PLoS One. 2022 Jul 20;17(7):e0270104. doi: 10.1371/journal.pone.0270104 (PMC9299332; doi:10.1371/journal.pone.0270104)
Supplement: S1 Checklist — (PDF) [file pone.0270104.s001.pdf]

Inclusivity in global research

PLOS' policy on inclusivity in global research aims to improve transparency in the reporting of research performed outside of researchers' own country or community and ensures that PLOS publications reporting global research adhere to high standards for research ethics and authorship. Authors of relevant research articles may be asked to complete the questionnaire below, which outlines ethical, cultural, and scientific considerations specific to inclusivity in global research. This questionnaire may be requested when researchers have travelled to a different country to conduct research, if research uses samples collected in another country, research with Indigenous populations or their lands, or if research is on cultural artefacts. Researchers travelling to another country solely to use laboratory equipment will not normally be required to complete the questionnaire. However, the questionnaire can be requested at the journal's discretion for any submission – if you have been requested to complete this questionnaire by the PLOS journal you submitted to, please do so.

Please complete the questionnaire below and include this as a Supporting Information file with your manuscript. Note that if your paper is accepted for publication, this checklist will be published with your article in the supporting information files. Please ensure that you reference the checklist in the main body of your manuscript. We suggest adding a subsection 'Inclusivity in global research' to your Methods section and adding the following sentence: "Additional information regarding the ethical, cultural, and scientific considerations specific to inclusivity in global research is included in the Supporting Information (SX Checklist)"

The questions have been designed to be applicable to a wide range of study types, and there are subsections for both human subjects research and non-human subjects research. If any of the questions are not relevant to your research please mark them as "N/A" as appropriate.

Ethical considerations, permits and authorship

This section is applicable to all research types.

Provide details as to who granted permissions and/or consent for the study to take place in the Methods section of your manuscript. This should include the names of **all** ethics boards, governmental organizations, community leaders or other bodies that provided approval for the study. If individuals provided approval refer to these people by their role or title but do not list their name(s).

Reported on page number: 5

If there were any deviations from the study protocol after approval was obtained please provide details of these changes in the Methods section of your manuscript.

Reported on page number: N/A

Did this study involve local collaborators that are residents of the country where the research was conducted or members of the community studied? If you do not have any authors from said communities, please provide an explanation for this below.

Yes, the study was conducted by a South African postgraduate as part of her PhD research (first author Jessica von der Meden), supervised by a South African lecturer (Dr Robyn Pickering). South African postgraduate Wendy Khumalo also co-authored the paper.

Everyone listed as an author should meet PLOS' criteria for authorship and all individuals who meet these criteria should be included in the author byline, rather than the acknowledgements. Authorship criteria is based on the International Committee of Medical Journal Editors (ICMJE) Uniform Requirements for Manuscripts Submitted to Biomedical Journals - for further information please see here: https://journals.plos.org/plosone/s/authorship.

Human subjects research (e.g. health research, medical research, cross-cultural psychology)

Did you obtain written informed consent from a representative of the local community or region before the research took place? How did you establish who speaks for the community? Details of written informed consent obtained from study participants should be reported separately in the Methods section of your manuscript.

N/A – no human subjects

How did members of the local community provide input on the aims of the research investigation, its methodology, and its anticipated outcome(s)?

N/A – no human subjects

When engaging with the local community, how did you ensure that the informed consent documents and other materials could be understood by local stakeholders?

N/A – no human subjects

Will the findings of the research be made available in an understandable format to stakeholders in the community where the study was conducted (e.g. via a presentation, summary report, copies of publications, etc.)? Please provide details of how this will be achieved.

N/A – no human subjects

Non-human subjects research using specimens/ animals collected as part of the study, or those housed in archival collections. Examples include archaeology, paleontology, botany and zoology.

Did the permission you obtained from a local authority to perform the study include an agreement on access to outputs and benefit sharing? This may include procedures to enable fair distribution of the benefits and resources arising from the research performed. Please include any details of Prior Informed Consent and Benefit Sharing Agreements obtained. These may be required by field-specific regulations, for example the Convention on Biological Diversity (CBD) and the associated Nagoya Protocol.

An agreement of the permit granted from the South African Heritage Resources Agency was that the study would support capacity development by providing research experience opportunities to South African postgraduate students. This was accomplished. The results reported in this particular paper are based in part on the work of two University of Cape Town postgraduate students (first author Jessica von der Meden and co-author Wendy Khumalo).

If the material used in your study was imported, please A) provide the year it was imported and B) indicate whether permits were obtained to import/export the materials used, C) provide details of any permits obtained. If this information is not available, please indicate this.

N/A – no archival material was imported/exported

If you used archival specimens, please state how the material used in your study was acquired by the institute it is held in and provide details of any permits obtained for the original excavations/ sample collection. If this information is not available, please indicate this.

N/A – no archival specimens were utilized

How was the potential cultural significance of the materials collected in your study to local communities considered in your research design? Were Indigenous peoples and/or local researchers and institutions involved with archaeological excavations / collection of specimens? If so, please provide a description of their involvement.

Ga-Mohana Hill is a culturally significant site and leaders of the local community, the Baga Motlhware Traditional Council, were consulted prior to the start our investigations. Recognising this significance, we adjusted our methods so as not to undermine the practices held there. We adopted a low-impact sampling approach; the geological samples (tufas) were carefully chosen after extensive survey of the 6 km area around the shelter, and we used a modified Makita hand drill with a small, custom-made core barrel to extract our samples with minimal disturbance to the features around the rock shelter. In this way, we were able to leave almost no visible trace of our work. While no archaeological artefacts or specimens were collected for this particular study, archaeological excavations, led by two co-authors on this paper (Dr Jayne Wilkins and Dr Benjamin Schoville) have been conducted at the site, and the archaeological findings inform some of the questions addressed in this paper. As with the tufa sample collection, the goal was minimal disturbance and so at the end of each season, archaeological excavations were completely back-filled and covered with sediment and no signage was erected so as not to effect any permanent modifications. Engagement with the local community is ongoing as we explore ways to integrate the cultural, archaeological and palaeoclimate value of Ga-Mohana Hill.

If your manuscript includes photographs of human remains please indicate whether authors obtained permission from descendants or affiliated cultural communities to do so.

N/A – no human remains were involved
