# Supplementary figures and images for: Tufas indicate prolonged periods of water availability linked to human occupation in the southern Kalahari
Source: PLoS One. 2022 Jul 20;17(7):e0270104. doi: 10.1371/journal.pone.0270104 (PMC9299332; doi:10.1371/journal.pone.0270104)

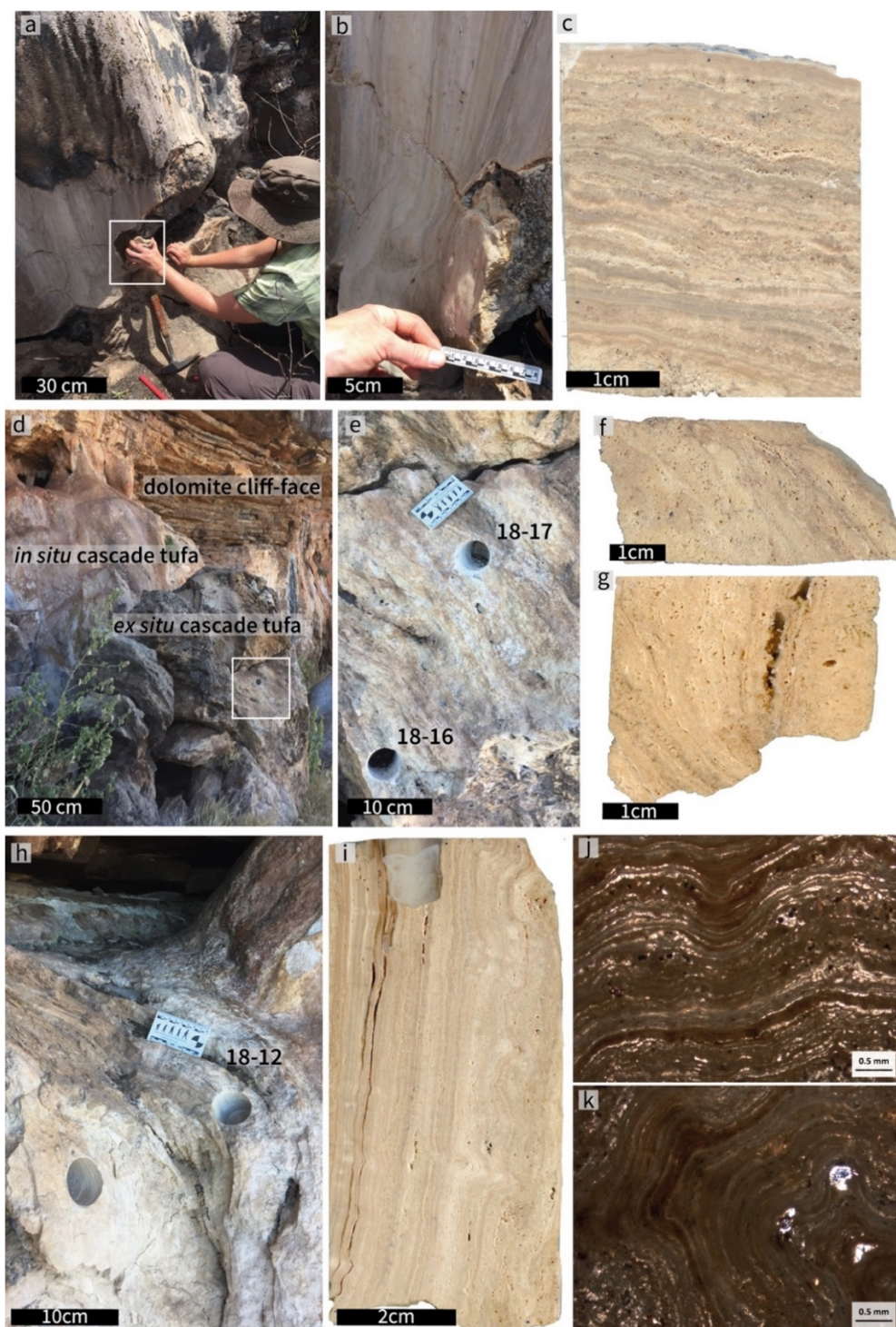

S1 Fig.

Supplement: S1 Fig — a,b) field context of sample 18–4; c) hand sample scan of 18–4 showing fine, undulating layers; d,e) field context of drill core samples 18–17 (f) and 18–16 (g); h) photograph of in-situ cascade tufa sampled with core drill; i) drill core sample 18–12; j,k) thin section photographs of sample 18–12 (in ppl) showing irregular, domal micritic laminae with lenses of microspar and micropores. (PDF) [file pone.0270104.s002.pdf]

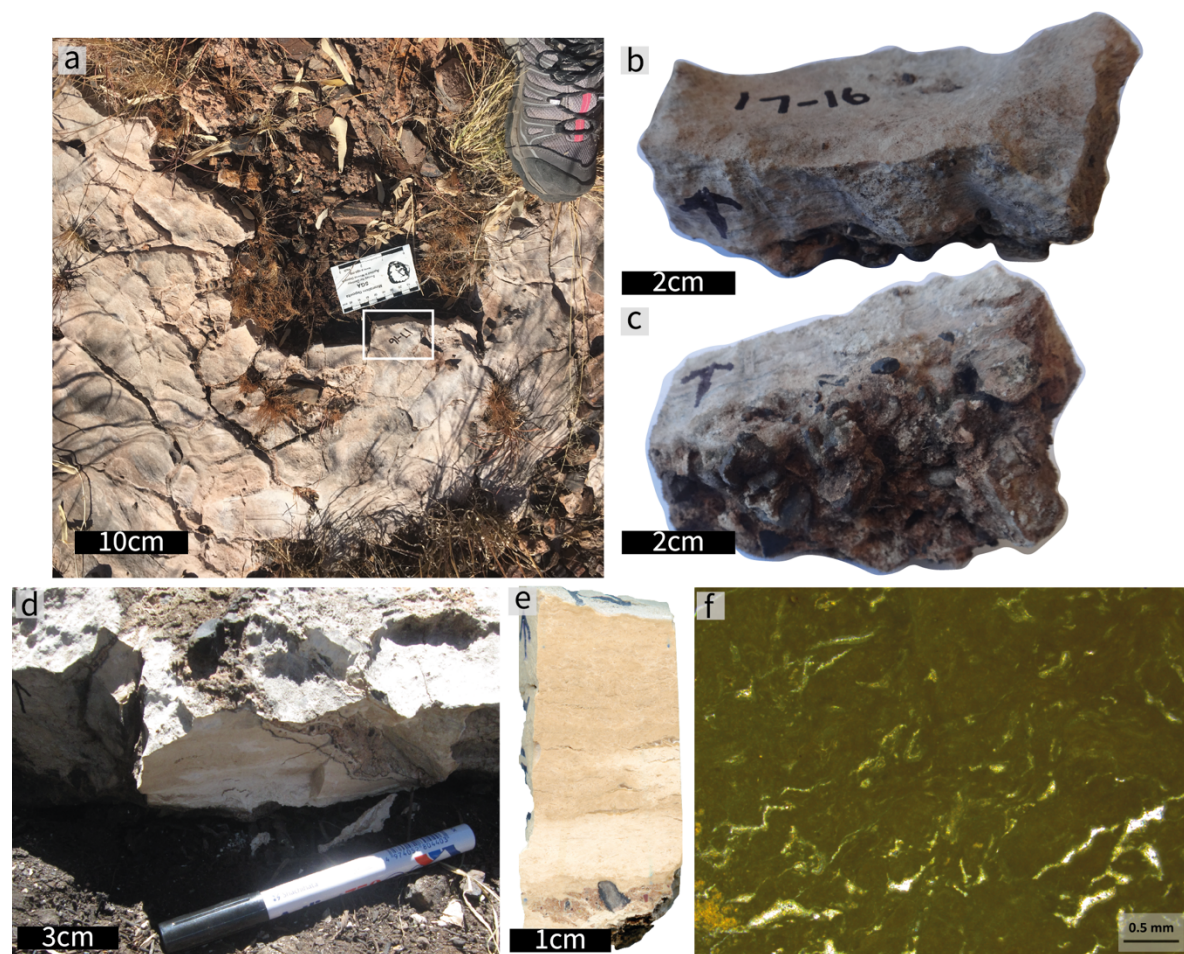

S2 Fig.

Supplement: S2 Fig — a) Field context of sample 17–16 showing circular configuration and surface desiccation cracks; b,c) hand sample photographs showing 4cm layer of carbonate and cemented clasts on the underside; d) field context of sample GHN-1; e) hand sample scan of GHN-1; c) photograph of thin section from GHN-1 showing aphanitic fabric of biomicrite with spar-filled filamentous cavities. (PDF) [file pone.0270104.s003.pdf]

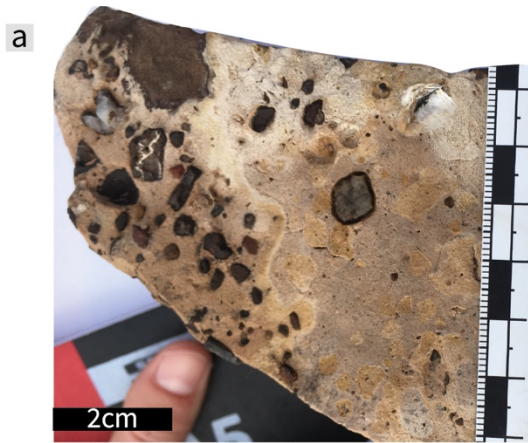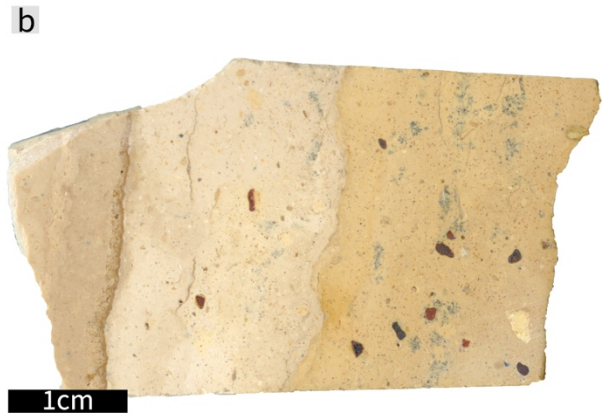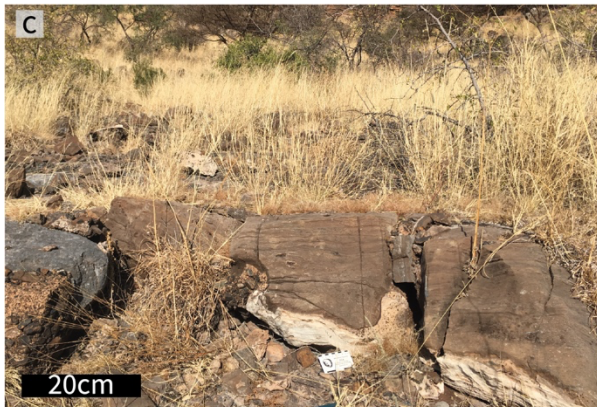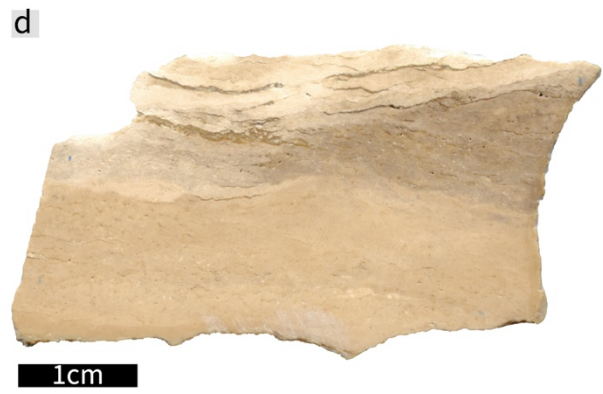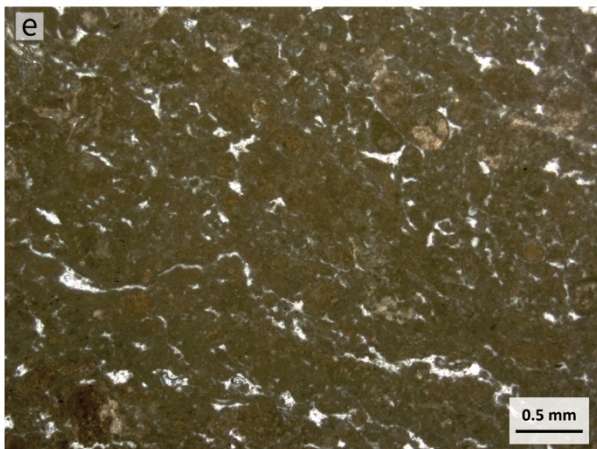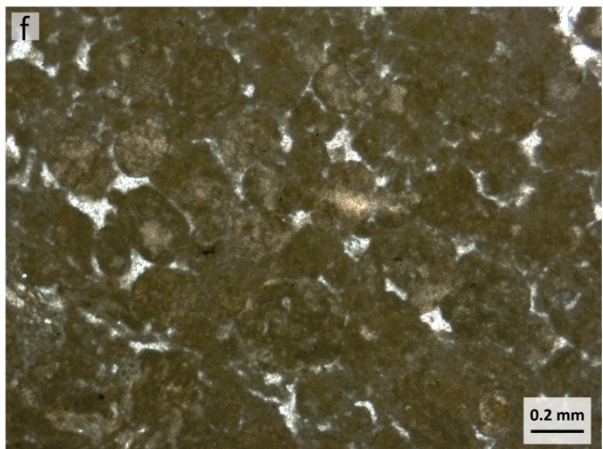

S3 Fig.

Supplement: S3 Fig — a) terrace breccia sample showing included detrital clasts and brecciated tufa clasts; b) hand sample scan of sample 18–7; c) field context and d) hand sample scan of sample 17–8 showing massive, dense micrite; e,f) thin section photographs of terrace sample 17–8 showing clotted fabric of peloidal micrite with microspar-filled void spaces. (PDF) [file pone.0270104.s004.pdf]

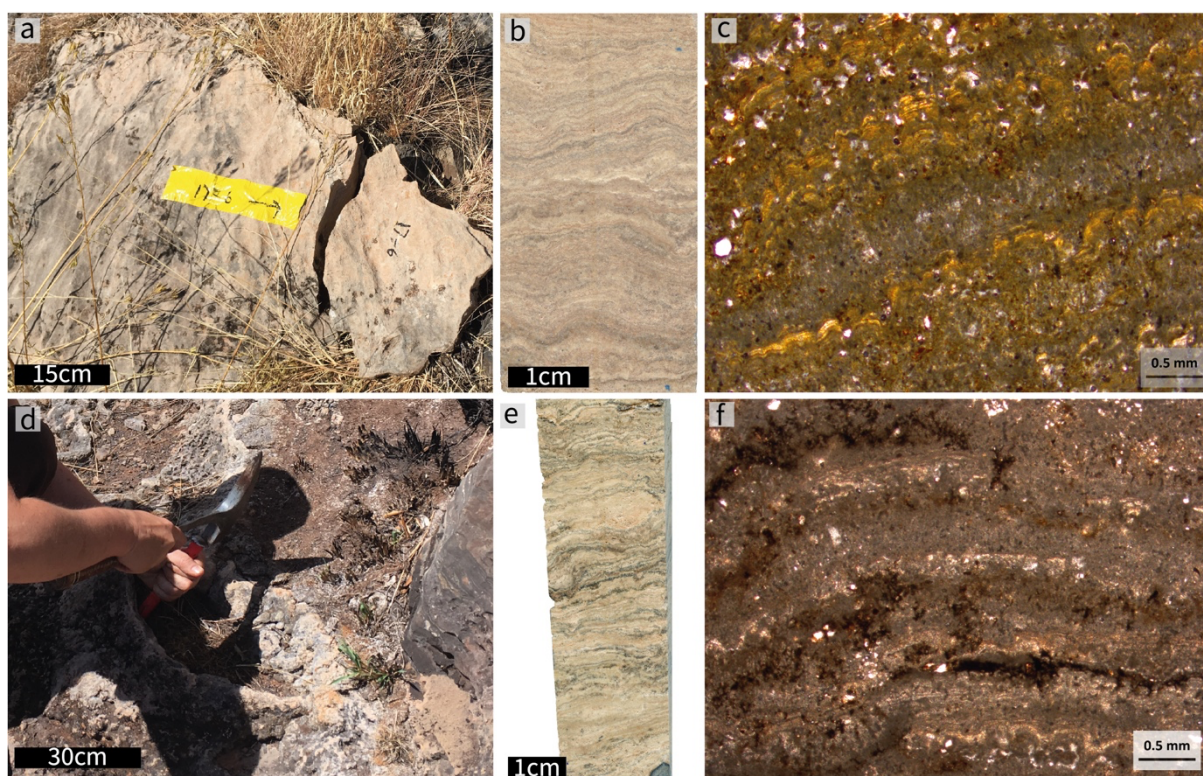

**S4 Fig.**

Supplement: S4 Fig — a) field photograph of sample 17–6; b) hand sample scan of sample 17–6 showing irregular, undulating and discontinuous layering; c) thin section photographs show stromatolite-type micrite crustal laminae alternating with chaotic microspar laminae with detrital and oxide inclusions (c) and discontinuous crinkly microspar laminae with overprinting of oxide precipitates (f). (PDF) [file pone.0270104.s005.pdf]

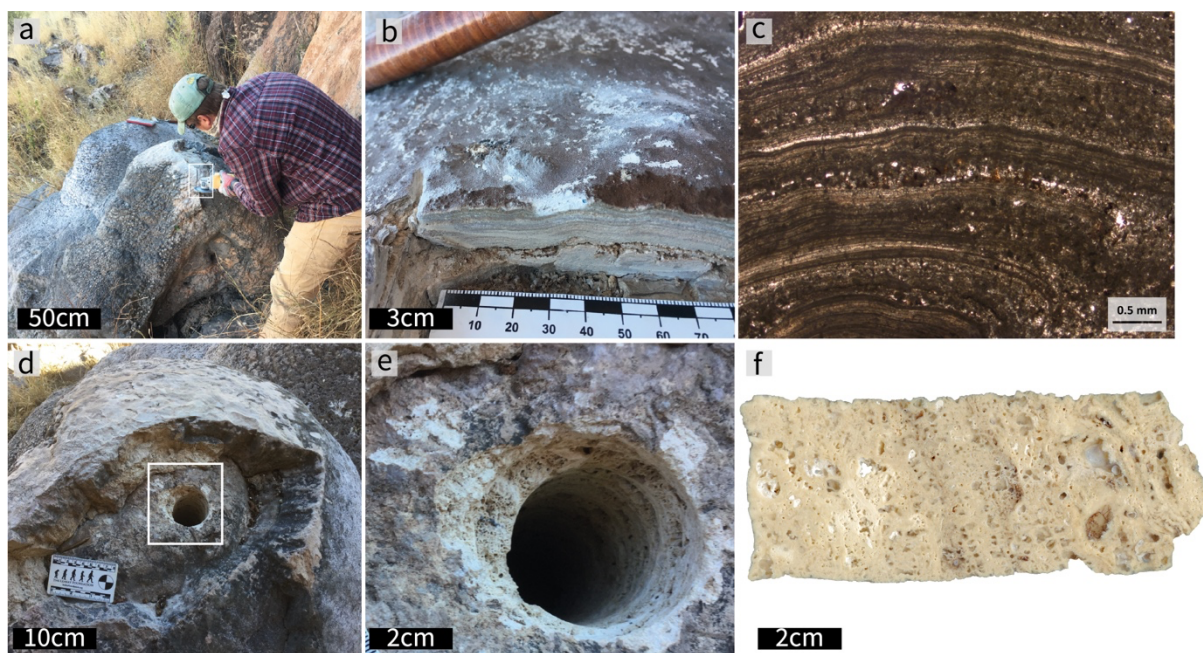

**S5 Fig.**

Supplement: S5 Fig — a) field context of tufa dome, sampled using an angle grinder; b) dense mm-scale layers alternating with irregular, porous and friable layers; c) photomicrograph of thin section from sample in (b) showing micro laminae; d,e) dome sampled with drill-core; f) hand sample of dome core showing large cavities and porous, reticulate framework. (PDF) [file pone.0270104.s006.pdf]

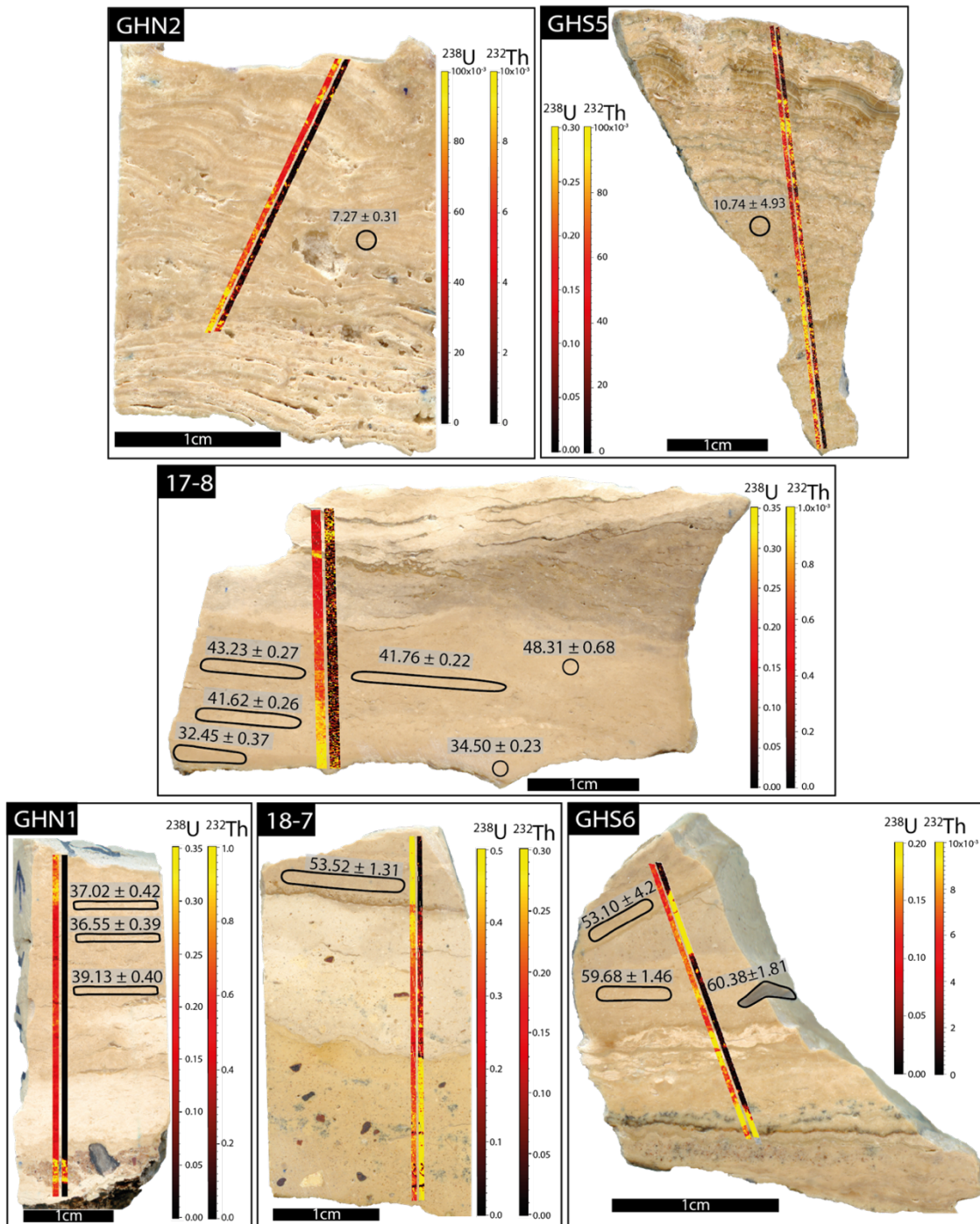

S6 Fig.

Supplement: S6 Fig — Concentrations in ppm shown in adjacent colour scales (warmer colour = higher concentration). Black circles represent approximate locations of subsamples drilled for U-Th dating prior to pre-screening, and oblong free-forms show exact locations at which subsamples were drilled for U-Th dating following LA-ICP-MS analysis. Ages associated with each subsample are given in thousands of years (ka) and are reported in Table 1. (PDF) [file pone.0270104.s007.pdf]

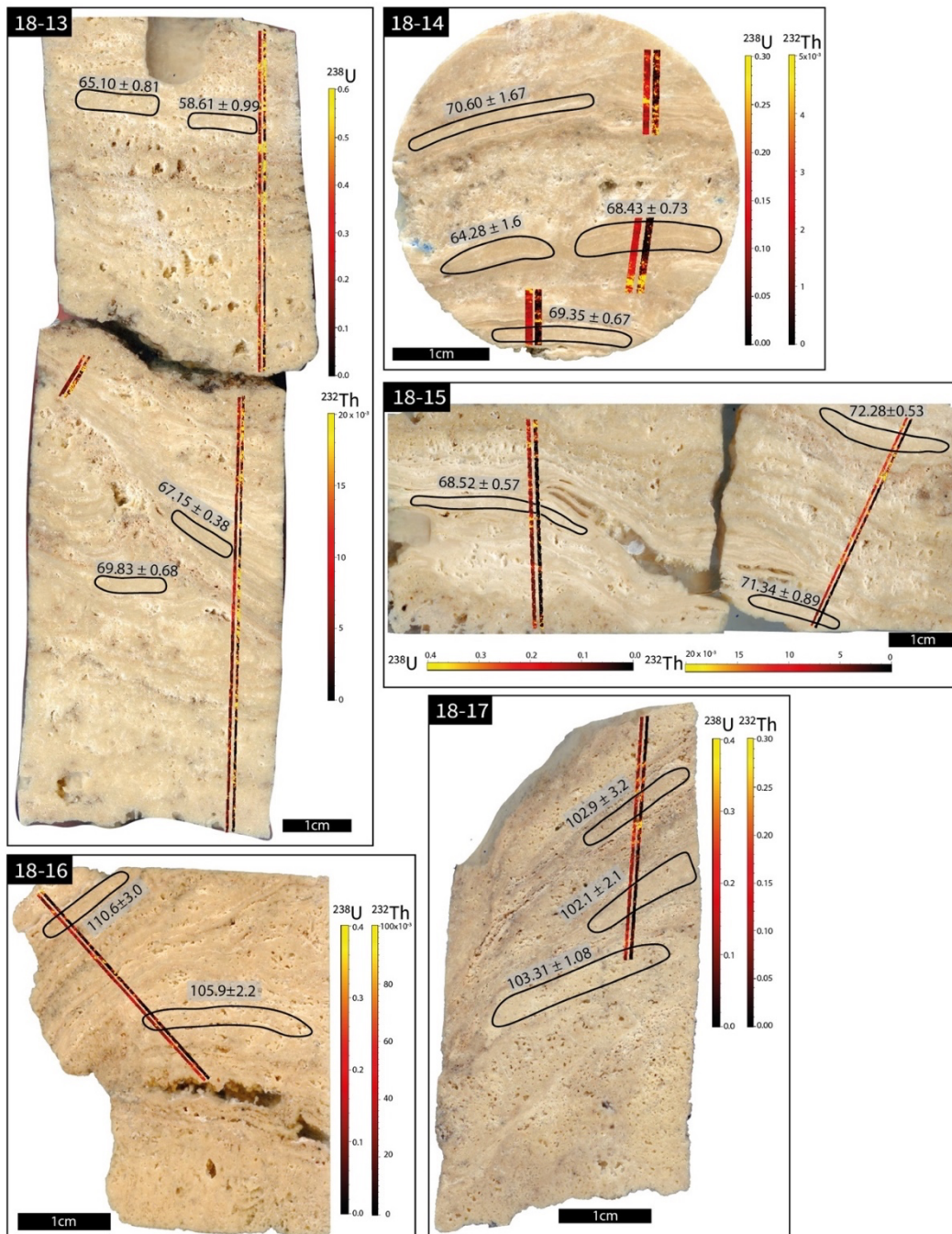

**S7 Fig.**

Supplement: S7 Fig — Concentrations shown in ppm in adjacent colour scales (warmer colour = higher concentration). Black circles represent approximate locations of subsamples drilled for U-Th dating prior to pre-screening, and oblong free-forms show exact locations at which subsamples were drilled for U-Th dating following LA-ICP-MS analysis. Ages associated with each subsample are given in thousands of years (ka) and are reported in Table 1. (PDF) [file pone.0270104.s008.pdf]

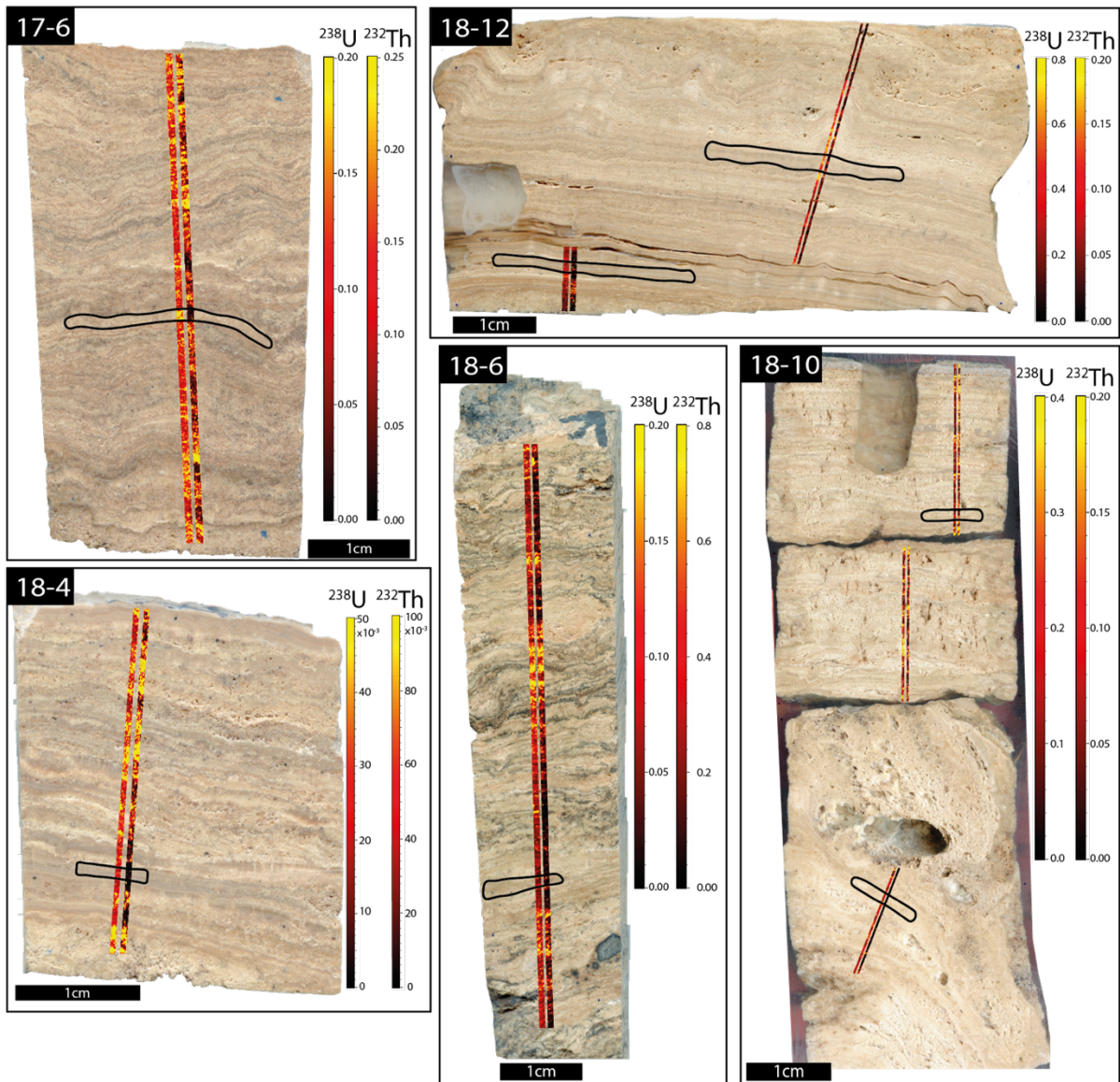

S8 Fig.

Supplement: S8 Fig — Black oblong outline represents material drilled for U-Th dating. Cascade samples (18–4 and 18–12), barrage samples (17–6 and 18–6) and dome core samples (18–10) overlain by LA-ICPMS 238U (left) and 232Th (right) element distribution maps. Concentrations in ppm shown in adjacent colour scales (warmer colour = higher concentration). (PDF) [file pone.0270104.s009.pdf]
