# Supplementary material for: Tufas indicate prolonged periods of water availability linked to human occupation in the southern Kalahari
Source: PLoS One. 2022 Jul 20;17(7):e0270104. doi: 10.1371/journal.pone.0270104 (PMC9299332; doi:10.1371/journal.pone.0270104)
Supplement: S1 Table — Samples with GH prefix collected in 2016, numerical prefix of other samples indicates the year they were collected (e.g. 17- = 2017). (PDF) [file pone.0270104.s010.pdf]

S1 Table.

Sample no.	Tufa type	U-Th age	2s	% error	**Laser ablation**	**Thin section**
GHN1	rim pool	39.126	0.398	1.0	X	X
		36.550	0.390	1.1		
		37.020	0.420	1.1		
GHN2	cascade	7.266	0.315	4.3	X	X
GHN3	dome	NO AGE				X
GHN4	dome	NO AGE				X
GHS5	cascade	10.738	4.932	45.9	X	X
GHS6	rim pool	60.379	1.809	3.0	X	X
		59.677	1.461	2.4		
		61.986	0.466	0.8		
		53.100	4.200	7.9		
17-6	barrage	NO AGE			X	X
17-8	terrace breccia	48.306	0.684	1.4	X	X
		34.503	0.231	0.7		
		41.760	0.220	0.5		
		43.230	0.270	0.6		
		41.620	0.260	0.6		
		32.450	0.370	1.1		
17-16	terrace breccia	not sampled				
18-4	cascade	NO AGE			X	X
18-6	barrage	NO AGE			X	X
18-7	terrace breccia	53.520	1.310	2.4	X	
18-8	dome core	not sampled				X
18-10	dome core	3.0	0.9	30	X	
18-11	cascade core	not sampled				X
18-12	cascade core	NO AGE			X	X
18-13	cascade core	58.610	0.990	1.7	X	
		65.040	0.810	1.2		
		67.150	0.380	0.6		
		69.830	0.680	1.0		
18-14	cascade core	68.430	0.730	1.1	X	X
		64.280	1.600	2.5		
		69.350	0.670	1.0		
		70.600	1.670	2.4		
18-15	cascade core	68.520	0.570	0.8	X	X
		71.340	0.890	1.2		
		72.280	0.530	0.7		
18-16	cascade core	110.600	3.000	2.7	X	X
		105.900	2.200	2.1		
18-17	cascade core	102.900	3.200	3.1	X	
		102.100	2.100	2.1		
		103.310	1.080	1.0		
