# Supplementary material for: Tufas indicate prolonged periods of water availability linked to human occupation in the southern Kalahari
Source: PLoS One. 2022 Jul 20;17(7):e0270104. doi: 10.1371/journal.pone.0270104 (PMC9299332; doi:10.1371/journal.pone.0270104)
Supplement: S2 Table — The samples are labelled according to the sequence they were collected in but presented in stratigraphic order. Errors on all isotope activity ratios are reported with 2σ uncertainty. All ages have been corrected to account for the effect of detrital Th assuming an estimate for initial 230Th/232Th of 1.5 ± 1.5, and calculated using the 230Th-238U decay constants of Cheng et al. [54] and equation 1 from Hellstrom [47]. (PDF) [file pone.0270104.s011.pdf]

S2 Table.

Sample ID	Lab number	Tufa type	^{238}U ng/g	$^{230}Th/^{238}U$	2s	$^{234}U/^{238}U$	2s	$^{232}Th/^{238}U$	2s	$^{230}Th/^{232}Th$	U-Th age (ka)	2s	% error
**18-10.2**	UME190515-620	dome	174	0.088	0.001	2.443	0.006	0.013409	0.000268	6.5	3.0	0.9	30
**GHN-2**	UMD160926-401	cascade	75	0.184	0.002	2.728	0.009	0.005083	0.000089	36.2	**7.3**	0.3	4.1
**GHS-5**	UMD160926-436	cascade	263	0.251	0.002	1.866	0.007	0.054496	0.000692	4.6	**10.7**	4.9	45.8
**17-8.1**	UMD180821-622	terrace	363	0.707	0.002	1.895	0.003	0.008000	0.000104	88.4	**48.3**	0.7	1.4
**17-8.2**	UMD180821-629	terrace	273	0.528	0.002	1.894	0.003	0.002214	0.000019	238.3	**34.4**	0.2	0.6
**17-8.3**	UME190515-202	terrace	321	0.621	0.002	1.896	0.005	0.000184	0.000004	3367.7	**41.8**	0.2	0.5
**17-8.4**	UME190515-252	terrace	298	0.641	0.003	1.903	0.005	0.000142	0.000003	4516.0	**43.2**	0.3	0.7
**17-8.5**	UME190515-255	terrace	307	0.618	0.003	1.895	0.005	0.000127	0.000003	4858.7	**41.6**	0.3	0.7
**17-8.6**	UME190515-535	terrace	459	0.503	0.004	1.899	0.006	0.002320	0.000046	216.6	**32.5**	0.4	1.2
**GHN-1**	UMD160926-523	rim pool	236	0.576	0.005	1.863	0.006	0.000805	0.000072	716.3	**39.1**	0.4	1.0
**GHN-1.2**	UME190515-307	rim pool	234	0.543	0.005	1.859	0.006	0.000080	0.000002	6818.5	**36.6**	0.4	1.1
**GHN-1.3**	UME190515-370	rim pool	240	0.551	0.005	1.868	0.007	0.000051	0.000001	10846.4	**37.0**	0.4	1.1
**GHS-6**	UMD160926-446	rim pool	212	0.864	0.007	1.914	0.007	0.020890	0.000507	41.3	**60.4**	1.8	3.0
**GHS-6.1**	UMD160926-438	rim pool	180	0.852	0.005	1.913	0.007	0.016957	0.000394	50.3	**59.7**	1.5	2.5
**GHS-6.2**	UME190515-547	rim pool	190	0.873	0.003	1.928	0.005	0.003473	0.000069	251.3	**61.9**	0.5	0.8
**GHS-6.3**	UME190516-210	rim pool	158	0.798	0.003	1.882	0.004	0.050119	0.001002	15.9	**53.1**	4.2	7.9
**18-7**	UME190515-298	terrace	847	0.749	0.003	1.833	0.005	0.014829	0.000297	50.5	**53.5**	1.3	2.4
**18-13.1**	UME190515-641	cascade	249	1.174	0.003	2.654	0.007	0.017183	0.000344	68.3	**58.6**	1.0	1.7
**18-13.2**	UME190515-647	cascade	226	1.313	0.003	2.742	0.007	0.014022	0.000280	93.6	**65.0**	0.8	1.2
**18-13.3**	UME190515-648	cascade	132	1.287	0.004	2.646	0.007	0.002075	0.000041	620.5	**67.2**	0.4	0.6
**18-13.4**	UME190515-651	cascade	195	1.483	0.004	2.933	0.008	0.011902	0.000238	124.6	**69.8**	0.7	1.0
**18-14.1**	UME190515-401	cascade	83	1.308	0.009	2.644	0.009	0.005376	0.000108	243.4	**68.4**	0.7	1.0
**18-14.2**	UME190515-405	cascade	139	1.219	0.006	2.551	0.008	0.026423	0.000528	46.1	**64.3**	1.6	2.5
**18-14.3**	UME190515-411	cascade	98	1.351	0.008	2.705	0.009	0.003077	0.000062	439.1	**69.4**	0.7	1.0

**18-14.4**	UME190515-502	cascade	180	1.481	0.006	2.876	0.008	0.031531	0.000631	47.0	**70.6**	1.7	2.4
**18-15.1**	UME190515-381	cascade	137	1.319	0.007	2.668	0.008	0.001688	0.000034	781.0	**68.5**	0.6	0.9
**18-15.2**	UME190515-383	cascade	95	1.317	0.011	2.587	0.010	0.001764	0.000035	746.9	**71.3**	0.9	1.3
**18-15.3**	UME190515-389	cascade	313	1.522	0.005	2.940	0.008	0.006626	0.000133	229.7	**72.3**	0.5	0.7
**18-17.1**	UME190516-208	cascade	154	2.176	0.006	3.194	0.006	0.074913	0.001498	29.0	**102.9**	3.2	3.1
**18-17.2**	UME190516-233	cascade	148	2.085	0.007	3.102	0.006	0.047194	0.000944	44.2	**102.1**	2.1	2.1
**18-17.3**	UME190516-235	cascade	142	2.217	0.006	3.289	0.006	0.023170	0.000463	95.7	**103.3**	1.1	1.1
**18-16.1**	UME190516-259	cascade	164	2.586	0.008	3.614	0.007	0.078583	0.001572	32.9	**110.6**	3.0	2.7
**18-16.2**	UME190516-283	cascade	177	2.404	0.007	3.476	0.007	0.054789	0.001096	43.9	**105.9**	2.2	2.1
