# Supplementary material for: Tufas indicate prolonged periods of water availability linked to human occupation in the southern Kalahari
Source: PLoS One. 2022 Jul 20;17(7):e0270104. doi: 10.1371/journal.pone.0270104 (PMC9299332; doi:10.1371/journal.pone.0270104)
Supplement: S3 Table — Errors on all isotope activity ratios are reported with 2σ uncertainty. Upper limit is defined as corrected age plus 2σ uncertainty. All ages have been corrected to account for the effect of detrital Th assuming an estimate for initial 230Th/232Th of 1.5 ± 1.5, and calculated using the 230Th-238U decay constants of Cheng et al. [54] and equation 1 from Hellstrom [47]. (PDF) [file pone.0270104.s012.pdf]

S3 Table.

Sample ID	Lab number	Tufa type	^{238}U ng/g	$^{230}Th/^{238}U$	2s	$^{234}U/^{238}U$	2s	$^{232}Th/^{238}U$	2s	$^{230}Th/^{232}Th$	U-Th age (ka)	2s	Estimated upper limit (ka)
**GHN3.1**	UMD160926-405	dome	209	0.238	0.004	2.396	0.007	0.202472	0.005325	1.2	-3.7	16.2	**<12.5**
**GHN3.2**	UMD160926-388	dome	149	0.283	0.007	2.263	0.007	0.263966	0.007649	1.1	-7.2	24.0	**<16.8**
**GHS4**	UMD160926-418	dome	207	0.242	0.003	1.75	0.007	0.314020	0.006247	0.8	-20.8	46.4	**<25.8**
**17-6.1**	UMD180821-621	barrage	196	0.295	0.002	1.949	0.003	0.315837	0.003331	0.9	-14.5	38.1	**<23.6**
**17-6.2**	UME190516-254	barrage	219	0.162	0.001	2.009	0.004	0.098911	0.001978	1.6	0.7	8.8	**<9.5**
**18-4**	UME190515-538	cascade	28	0.199	0.006	1.752	0.01	0.170880	0.003418	1.2	-4.6	19.1	**<14.5**
**18-6**	UME190516-328	barrage	68	0.601	0.004	1.739	0.004	0.835852	0.016717	0.7	-	-	***infinity***
**18-10.1**	UME190515-617	dome	86	0.484	0.003	2.393	0.007	0.627906	0.012558	0.8	-	-	***infinity***
**18-12.1**	UME190516-286	cascade	379	7.29	0.017	2.296	0.005	1.371873	0.027437	5.3	-1532.0	11145.3	***no age***
**18-12.2**	UME190516-295	cascade	414	0.223	0.002	2.49	0.005	0.075426	0.001509	3	5.1	5.1	**<10.2**
