# Supplementary material for: Tufas indicate prolonged periods of water availability linked to human occupation in the southern Kalahari
Source: PLoS One. 2022 Jul 20;17(7):e0270104. doi: 10.1371/journal.pone.0270104 (PMC9299332; doi:10.1371/journal.pone.0270104)
Supplement: Attachment — Submitted filename: PONE-D-22-02819_reviewer_EC.pdf [file pone.0270104.s013.pdf]

PLOS ONE

Tufas indicate decoupling of water availability and human occupation in the southern Kalahari

--Manuscript Draft--

**Manuscript Number:**	PONE-D-22-02819
**Article Type:**	Research Article
**Full Title:**	Tufas indicate decoupling of water availability and human occupation in the southern Kalahari
**Short Title:**	Water availability and human occupation in the southern Kalahari
**Corresponding Author:**	Jessica von der Meden<br>University of Cape Town<br>Cape Town, Western Cape SOUTH AFRICA
**Keywords:**	U-Th dating; tufa; Kalahari; palaeoclimate; climate; human evolution; Ga-Mohana Hill
**Abstract:**	Detailed, well-dated palaeoclimate and archaeological records are critical for understanding the impact of environmental change on human evolution. Ga-Mohana Hill, in the southern Kalahari, South Africa, preserves a Pleistocene archaeological sequence. Relict tufas at the site are evidence of past flowing streams, waterfalls, and shallow pools. Here, we report an extensive dating programme of the tufas. Using laser ablation screening to target material suitable for uranium-thorium dating, we obtained 33 ages covering the last 110 thousand years (ka). We identify four tufa formation episodes at 114-100 ka, 73-48 ka, 44-32 ka, and 15-2 ka. Three tufa episodes are coincident with archaeological units at Ga-Mohana Hill, dated to ~105 ka, ~31 ka, and ~15 ka. Together with nearby palaeoenvironmental and archaeological records at Wonderwerk Cave and Kathu Pan, we argue that in the southern Kalahari, from ~240 ka to ~71 ka wet phases and human occupation are coupled, but after ~71 ka they are decoupled.
**Order of Authors:**	Jessica von der Meden
	Robyn Pickering
	Benjamin J. Schoville
	Helen Green
	Rieneke Weij
	John Hellstrom
	Alan Greig
	Jon Woodhead
	Wendy Khumalo
	Jayne Wilkins
**Additional Information:**	
**Question**	**Response**
**Financial Disclosure**<br><br>Enter a financial disclosure statement that describes the sources of funding for the work included in this submission. Review the submission guidelines for detailed requirements. View published research articles from *PLOS ONE* for specific examples.	Funding for this project was provided by the Department of Science and Technology National Research Foundation (DST-NRF, South Africa) Centre of Excellence in Palaeosciences through student bursaries (JvdM and WK) and Operations grants - CoE2017-065, COE2018-05OP, COE2019-OP17 and COE2018-10OP (JWi and RP); the National Research Foundation (NRF, South Africa) African Origins Platform Grant - AOP150924142990 (RP), NRF (South Africa) Competitive Programme for Rated Researchers -120806 (RP), National Geographic Society - Waitt Grant (BJS), University of Cape Town VC2030 funding (RP), NRF (South Africa) Research Development Grant for Y-rated Researchers (JWi), and Australian Research Council

This statement is required for submission and **will appear in the published article** if the submission is accepted. Please make sure it is accurate.<br><br>**Unfunded studies**<br>Enter: *The author(s) received no specific funding for this work.*<br><br>**Funded studies**<br>Enter a statement with the following details:<br>• Initials of the authors who received each award<br>• Grant numbers awarded to each author<br>• The full name of each funder<br>• URL of each funder website<br>• Did the sponsors or funders play any role in the study design, data collection and analysis, decision to publish, or preparation of the manuscript?<br>• **NO** - Include this sentence at the end of your statement: *The funders had no role in study design, data collection and analysis, decision to publish, or preparation of the manuscript.*<br>• **YES** - Specify the role(s) played.<br><br>* typeset	Discovery Early Career Research Award - DE 190100160 (JWi). The funders had no role in study design, data collection and analysis, decision to publish, or preparation of the manuscript.
**Competing Interests**<br><br>Use the instructions below to enter a competing interest statement for this submission. On behalf of all authors, disclose any competing interests that could be perceived to bias this work—acknowledging all financial support and any other relevant financial or non-financial competing interests.<br><br>This statement is required for submission and **will appear in the published article** if the submission is accepted. Please make sure it is accurate and that any funding sources listed in your Funding Information later in the submission form are also declared in your Financial Disclosure statement.<br><br>View published research articles from	The authors have declared that no competing interests exist.

PLOS ONE for specific examples.<br><br>**NO authors have competing interests**<br><br>Enter: *The authors have declared that no competing interests exist.*<br><br>**Authors with competing interests**<br><br>Enter competing interest details beginning with this statement:<br><br>*I have read the journal's policy and the authors of this manuscript have the following competing interests: [insert competing interests here]*<br><br>* typeset	
**Ethics Statement**<br><br>Enter an ethics statement for this submission. This statement is required if the study involved:<br><br>• Human participants<br>• Human specimens or tissue<br>• Vertebrate animals or cephalopods<br>• Vertebrate embryos or tissues<br>• Field research<br><br>Write "N/A" if the submission does not require an ethics statement.<br><br>General guidance is provided below. Consult the submission guidelines for detailed instructions. **Make sure that all information entered here is included in the Methods section of the manuscript.**	N/A

Powered by Editorial Manager® and ProduXion Manager® from Aries Systems Corporation

**Format for specific study types**<br><br>**Human Subject Research (involving human participants and/or tissue)**<br>• Give the name of the institutional review board or ethics committee that approved the study<br>• Include the approval number and/or a statement indicating approval of this research<br>• Indicate the form of consent obtained (written/oral) or the reason that consent was not obtained (e.g. the data were analyzed anonymously)<br><br>**Animal Research (involving vertebrate animals, embryos or tissues)**<br>• Provide the name of the Institutional Animal Care and Use Committee (IACUC) or other relevant ethics board that reviewed the study protocol, and indicate whether they approved this research or granted a formal waiver of ethical approval<br>• Include an approval number if one was obtained<br>• If the study involved *non-human primates*, add *additional details* about animal welfare and steps taken to ameliorate suffering<br>• If anesthesia, euthanasia, or any kind of animal sacrifice is part of the study, include briefly which substances and/or methods were applied<br><br>**Field Research**<br><br>Include the following details if this study involves the collection of plant, animal, or other materials from a natural setting:<br>• Field permit number<br>• Name of the institution or relevant body that granted permission	
**Data Availability**<br><br>Authors are required to make all data underlying the findings described fully available, without restriction, and from the time of publication. PLOS allows rare exceptions to address legal and ethical concerns. See the PLOS Data Policy and FAQ for detailed information.	Yes - all data are fully available without restriction

A Data Availability Statement describing where the data can be found is required at submission. Your answers to this question constitute the Data Availability Statement and **will be published in the article**, if accepted.<br><br>**Important:** Stating 'data available on request from the author' is not sufficient. If your data are only available upon request, select 'No' for the first question and explain your exceptional situation in the text box.<br><br>Do the authors confirm that all data underlying the findings described in their manuscript are fully available without restriction?	
**Describe where the data may be found in full sentences. If you are copying our sample text, replace any instances of XXX with the appropriate details.**<br><br>• If the data are **held or will be held in a public repository**, include URLs, accession numbers or DOIs. If this information will only be available after acceptance, indicate this by ticking the box below. For example: *All XXX files are available from the XXX database (accession number(s) XXX, XXX.).*<br>• If the data are all contained **within the manuscript and/or Supporting Information files**, enter the following: *All relevant data are within the manuscript and its Supporting Information files.*<br>• If neither of these applies but you are able to provide **details of access elsewhere**, with or without limitations, please do so. For example:<br><br>*Data cannot be shared publicly because of [XXX]. Data are available from the XXX Institutional Data Access / Ethics Committee (contact via XXX) for researchers who meet the criteria for access to confidential data.*<br><br>*The data underlying the results presented in the study are available from (include the name of the third party*	All relevant data are within the manuscript and its Supporting Information files.

and contact information or URL).<br>• This text is appropriate if the data are owned by a third party and authors do not have permission to share the data.<br><br>* typeset	
Additional data availability information:	

Powered by Editorial Manager® and ProduXion Manager® from Aries Systems Corporation

Manuscript

Click here to access/download;Manuscript;von der Meden et al PLOS ONE_Jan2022.docx

Tufas indicate decoupling of water availability and human occupation in the southern Kalahari

Jessica von der Meden[1,2]*, Robyn Pickering[1,2], Benjamin J. Schoville[2,4], Helen Green[5], Rieneke Weij[5], John Hellstrom[5], Alan Greig[5], Jon Woodhead[5], Wendy Khumalo[1,2], Jayne Wilkins[2,3]

[1]Department of Geological Sciences, University of Cape Town, Rondebosch, South Africa

[2]Human Evolution Research Institute, University of Cape Town, Rondebosch, South Africa

[3]Australian Research Centre for Human Evolution, Environmental Futures Research Institute, Griffith University, Nathan, Australia

[4]School of Social Science, University of Queensland, St Lucia, Queensland, Australia

[5]School of Geography, Earth and Atmospheric Sciences, The University of Melbourne, Parkville, Victoria, Australia

*Corresponding author

Email: vdrjes001@myuct.ac.za (JvdM)

Abstract

Detailed, well-dated palaeoclimate and archaeological records are critical for understanding the impact of environmental change on human evolution. Ga-Mohana Hill, in the southern Kalahari, South Africa, preserves a Pleistocene archaeological sequence. Relict tufas at the site are evidence of past flowing streams, waterfalls, and shallow pools. Here, we report an extensive dating programme of the tufas. Using laser ablation screening to target material suitable for uranium-thorium dating, we obtained 33 ages covering the last 110 thousand years (ka). We identify four tufa formation episodes at 114-100 ka, 73-48 ka, 44-32 ka, and 15-2 ka. Three tufa episodes are coincident with archaeological units at Ga-Mohana Hill, dated to ~105 ka, ~31 ka, and ~15 ka. Together with nearby palaeoenvironmental and archaeological records at Wonderwerk Cave and Kathu Pan, we argue that in the southern Kalahari, from ~240 ka to ~71 ka wet phases and human occupation are coupled, but after ~71 ka they are decoupled.

Introduction

A key question in human origins research is how climate change impacted early *Homo sapiens* population distributions across Africa. It has been hypothesized that humans did not always have the capacity to survive in arid environments[1, 2], that early human distributions were modulated by distance to[3] and availability of water [4], that people were largely restricted to wetter refugia during glacial periods[5, 6], and that the occupation of arid regions was coincident with interglacial periods[7, 8]. The Kalahari Basin in the interior of southern Africa is characterized by low precipitation and high evaporation, which results in low surface water and arid/semi-arid conditions today[9]. However, there is evidence that the southern Kalahari was wetter during some periods in the Pleistocene[10]. For example, multiple paleoenvironmental proxies demonstrate climatic shifts through the Pleistocene and Holocene at Wonderwerk Cave[11-16], and at Kathu Pan,

several wet periods between ~160-22 ka have been identified based on sedimentary analyses [17, 18]. Evidence for wetter conditions at Ga-Mohana Hill ~110-105 ka has also been reported[19]. As a semi-arid region that has experienced significant climatic fluctuations with abundant records of both palaeoenvironment and archaeology, the southern Kalahari Basin provides a unique opportunity to further explore early human-environment interactions[20]. Previous studies reveal significant complexities even at the intra-regional scale, however, due in part to the different types of proxies with variable resolutions and the variety of forcing factors at play[10]. They also reveal a complex relationship between palaeoenvironmental conditions and evidence for human occupation[20, 21]. To assess the response of *Homo sapiens* to changes in climate and environments, well-dated and integrated records of past environments and human behaviour are required. We report one such record here that spans the last 110 thousand years.

Ga-Mohana Hill is a double-humped hillside comprised of stepped Palaeoproterozoic dolomites and capped by a layer of banded iron formation[22]. It is situated on the eastern flank of the north-south trending Kuruman Hills which outcrop on the Ghaap Plateau, an elevated region in the Northern Cape province of South Africa (Fig 1). Today the area is characterised as semi-arid, with seasonal mean annual precipitation of ~300-400 mm during the austral summer months[23]. Recent archaeological excavations at Ga-Mohana Hill North Rockshelter have yielded a Middle Stone Age assemblage of artefacts that provides early evidence for innovation and behavioural complexity in this region at 105±3.3 ka[19, 24]. Stratified above are younger deposits dated by OSL to 31±1.8 ka and 15±0.8 ka[24]. The hillside has abundant carbonate deposits, identified as tufa, i.e., ambient temperature, freshwater calcium carbonate precipitates. Tufas are direct evidence for the presence of water on the landscape and are amenable to radiometric dating methods, making them valuable archives of changes in environmental conditions[25-30]. In this study, we present macro- and micromorphological analyses of the Ga-Mohana tufas to assess their depositional context. Tufas

are challenging materials for dating due to detrital contamination and generally low uranium concentrations[31, 32] and so samples were pre-screened using laser ablation inductively coupled plasma mass spectrometry (LA-ICP-MS) to target optimal zones for study. This method has been used previously for dating speleothems[33], but to the best of our knowledge, this is the first time it has been applied to tufas. We obtained 33 U-Th age estimates and identify four wet periods, providing a record of localized climate change linked to a dated record of human occupation.

Fig 1. Map of South Africa with the location of Ga-Mohana Hill (GHN) and key palaeoenvironmental and Middle Stone Age sites discussed in the text. Dashed lines demarcate summer and winter rainfall zone boundaries (SRZ, WRZ), middle area experiences year-round rainfall (YRZ). Inset map shows the approximate extent of the Kalahari Basin in southern Africa and the location of the region of interest in relation to it.

Materials and Methods

Fieldwork and tufa sample collection

Ga-Mohana Hill has spiritual significance for the local communities, with visits to the shelter deliberate and rare[34]. Out of respect for this and as part of our on-going engagement with these communities, we adopted a low-impact sampling approach, with targeted samples carefully chosen after extensive survey of the 6 km area around the shelter. During this pedestrian survey, the field occurrences, positions and types of tufa were identified and mapped using a roaming Geographic Positioning System. A total of twenty-nine tufa hand samples were collected from the ~ 1 km^2 Ga-Mohana hillside sampling all five tufa morphologies recognised. Eighteen hand samples were collected using a geological hammer, mallet and chisel, marking the way-up on each sample with an arrow using permanent marker. Material sampled from the outer layer of cascade tufas returned

Holocene ages. Subsequent sampling deliberately targeted the stratigraphically older layers, closest to the host rock dolomite, in order to try and constrain the onset of preserved tufa formation. We used a modified Makita cordless hand drill fitted with Pomeroy Model SW-3 Miniature Water Swivel and a custom made Pomerory 1.5" ID diamond-tipped core barrel. A total of eleven small cores were collected, 8 cm in length on average, from *in-situ* mound tufas, and both *in-situ* and *ex-situ* cascade tufas (S1 Table, S1-S5 Figs). The cores were set in epoxy resin and then halved lengthways with a diamond rock saw and polished. Thin sections were made from a sub-set of fourteen samples, representative of all the morphology types, for characterisation using a Zeiss AXIO polarising light microscope (S1 Table, S1-S5 Figs).

Laser ablation-Inductively Coupled Plasma-Mass Spectrometer (LA-ICP-MS) pre-screening of U and Th concentrations and distributions

The aphanitic micrite layers free from detritus and inclusions, identified in thin section, were primary targets for U-Th dating. However, these layers tend to be fine, undulating and laterally variable, and so while visual evaluation of the tufas is an important first step in identifying suitable material to target for U-Th dating, it is not sufficient considering the complexity of the tufas on a microscale. We employed an additional pre-screening step, using laser ablation inductively coupled plasma mass spectrometry (LA-ICP-MS), to measure and image the U and Th concentrations and distributions along transects within the tufa samples. This allowed us to target layers with sufficiently high levels of ^{238}U, and low levels of ^{232}Th, i.e. detrital thorium, as these are the best targets for producing reliable age data[35].

Tufa U and Th concentrations and distributions were collected for 16 samples using laser-ablation with an Applied Spectra RESOlution SE 193nm ArFexcimer laser-ablation system coupled to an Agilent 7700x Quadrupole ICP-MS at the University of Melbourne, following the protocols

outlined in Woodhead et al.[36]. High-resolution images (3200 dpi) of the samples were captured using a flat-bed scanner, used to reference the co-ordinate system of the laser cell using GeoStar software (Norris Software). Between 6 and 12 parallel lines per sample, set 62µm apart, were chosen perpendicular to the growth layers. Pre-ablation was performed twice using a 60µm spot size and stage translation speed of 150µm/s.

Trace element data for the following elements: Mg, Al, Mn, Fe, Zn, Sr, Ba, Pb, Th and U, were collected with a 60µm spot at a stage translation speed of 75µm/s, pulse rate of 10Hz, and laser fluence of ~2-3 Jcm^{-2}. NIST SRM 612 was used for calibration, with ^{43}Ca as an internal standard, and an estimated precision of ca <5%. NIST SRM 610 and JCp-1, a powdered coral standard, were also analysed. The raw mass spectrometry data was reduced using the Iolite software package[37, 38]. Element distribution maps were generated in order to visualise the spatial arrangement of the various trace elements through the samples[39] (S6-S8 Figs). Layers with elevated levels of ^{238}U accompanied by little to no ^{232}Th were targeted for U-Th sampling. These samples were then chemically processed for U-Th dating following protocols described in Hellstrom[35, 40].

U-Th dating of tufa

Guided by the laser ablation results, layers with sufficiently high uranium (^{238}U > 0.1ppm) and low thorium concentrations (^{232}Th < 0.01ppm) were selected for U-Th analysis. A subset of 43 samples (S2 and S3 Tables, S6-S8 Figs), each with a mass of 60 ± 10 mg, were drilled from 16 tufa samples using a Dremel hand-held hobby drill and 1 mm carbide micro-drill bit. Powdered samples were dissolved in 1.5M HNO_3, spiked with a mixed ^{236}U-^{233}U-^{229}Th tracer equilibrated on a hotplate overnight. U and Th were separated from the calcite matrix using Eichrom TRU-spec selective ion exchange resin following established protocols[40]. The U-Th solution was dissolved in a mixture of dilute nitric and hydrofluoric acid and introduced to the Nu Instruments Plasma Multi Collector-

Inductively Coupled Plasma-Mass Spectrometer via an autosampler[40, 41]. Isotope-ratio measurements for $^{230}Th/^{238}U$ and $^{234}U/^{238}U$ were calculated using an internally standardised parallel ion-counter procedure and calibrated against the secular equilibrium standard, HU-1. Reproducibility was monitored using a second in-house standard (YB-1). An a *priori* estimate of 1.5 ± 1.5 for the initial $^{230}Th/^{232}Th$ was applied to all the samples in order to correct for the inherent detrital component[35]. With this initial value and its uncertainty, corrected ages for all samples were calculated using Monte Carlo iterations to solve equation 1 of Hellstrom[35] and the half-life values of ^{234}U and ^{230}Th as reported in Cheng et al.[42]. The final age uncertainty is reported in 2σ for which the uncertainties of the measured activity ratios as well as the assumed initial $^{230}Th/^{232}Th$ are fully propagated.

Results

Tufa macro and micromorphology

The Ga-Mohana tufa system comprises five morphological components: cascades, rim pools, barrages, domes and terrace breccias (Fig 2). The bedrock lithology of the Ghaap Plateau, which is comprised of the Palaeoproterozoic dolomites of the Campbellrand-Malmani Subgroup[22], has undergone extensive karstification. Coupled with cross-cutting dolerite dykes, this has resulted in large groundwater compartments that are important aquifers for the region[43, 44]. Groundwater resurgence at active springs in the area, such as the Eye of Kuruman, in Kuruman (Fig 1) are a testament to this vast underground drainage network[44]. The presence and movement of these groundwaters through the dolomite host rock is a vital precursor to the formation of the tufas at Ga-Mohana Hill.

Fig 2. Tufa depositional environment context and representative photographs of each of the tufa morphologies identified on the Ga-Mohana hillside. (**A**) Schematic profile sketch of Ga-Mohana Hill North Rockshelter (not drawn to scale) illustrating the series of tufa deposits and the archaeological excavation. The excavation layers dated via OSL: DBSR = ~105 ka; OAS = ~31 ka and DBGS = ~15 ka; (**B**) cliff cascade; (**C**) step-front cascade; (**D**) sinuous rim-pool edge; (**E**) barrage tufa; (**F**) terrace breccia; (**G**) tufa dome.

Tufa cascades are observed across the ~1 km^2 hillside. Large (3-5m) cascades cover the tall cliffs on either side of the rock shelters. Smaller cascades bulge outward from the fronts of the dolomite steps above and below the shelters (Fig 2, S1 Fig). These cascade tufas are point-sourced, appearing to have formed from water flowing out of the dolomite bedding planes. Below the step-front cascades, sinuous tufa rims edge the flat, transverse sections of the dolomite steps. These are evidence of terraced, shallow pools, likely formed from excess water ponding below the cascades. The areas behind the rims are filled with lightly compacted sediment and debris. Curved, down-hill sloping barrage tufas, characterised by knobbly, coralloid surfaces, sit below the rim pool edges formed when water overflowed from the pools above. Meandering channels scoured in the dolomite, observed above the rock shelter, are evidence of palaeostreams and indicate periods of substantial and prolonged water flow.

The rockshelters and the tall cliffs adjacent to them mark a break in the hillside. At this point, cone-shaped tufa ‘noses’ jut out over the lip of the rock shelters and are spread along the overhang (Fig 2). These are interpreted as remnants of moss curtains and align with large hemispheric dome tufas below. The domes trace the dripline of the overhanging shelters and occur along the base of the cliffs adjacent to the rock shelters; they appear to be sourced from dripping and splashing waters channelled via the noses above. The internal rockshelter walls are covered with clusters of small

stalactitic features and calcitic crusts. Below the shelter, surface-cementations of sub-angular detrital clasts of variable sizes (0.5-20 cm) of banded ironstone and dolomite fragments occur as benches, pavements, or patches of carbonate-cemented hill-slope material on the sub-horizontal terraces between dolomite steps. In rare instances, stone artefacts are also included.

Microscale observations of the tufas reveal that, regardless of depositional setting, the tufas are composed of a few simple petrographic components: micrite, microspar, and sparite (S1-S5 Figs). Detrital clasts (quartz) and iron and manganese oxides are present to varying degrees and tend to be concentrated along thin layers in the tufas. This suggests periods of non-deposition of tufa. The variable organisation of these petrographic components within each sample results in distinct fabrics, classed as laminar, peloidal, aphanitic, and chaotic, following the scheme devised by Manzo et al. [45]. A significant biological component is evidenced by stromatolitic structures (S1 Fig), clotted micrite (S3 Fig), and primary cavities (S5 Fig).

The various tufa morphologies at Ga-Mohana Hill each represents an individual sub-environment, and together they form a continuum of linked deposits. This depositional environment was characterised by water emerging from bedding planes in the dolomite, flowing down the hillside via multiple divergent pathways, creating cascades on the step-fronts of the dolomite steps, generating waterfalls and moss curtains over the rock shelters, and feeding shallow pools on the flat terraces. The terrace breccia deposits hint at periods of high energy flow (e.g., flash flooding) to transport and cement substantial talus scree downslope.

U-Th Chronology

Of 21 sampled tufas, 16 were subjected to the LA-ICP-MS pre-screening process (S1 Table, S6-S8 Figs). The ^{238}U concentration in the tufas is consistently low (range = 0.1 to 0.6 ppm; mean =

0.2 ppm). The ^{232}Th concentrations were generally lower than the ^{238}U concentrations, with most samples reflecting a wide range in ^{232}Th concentration, between 1-100 ppb. In many instances, elevated ^{232}Th corresponds with visually discernible detrital material (S8 Fig).

Layers of tufa with elevated levels of ^{238}U accompanied by minimal ^{232}Th were targeted for U-Th sampling. Out of 43 sub-samples drilled from 18 tufa samples, we obtained 33 U-Th ages from 12 tufas (Table 1, S2 Table). Cascade, rim pool and terrace tufas exhibited high success rates; 86% of cascade samples (18 of 21), 100% of rim pools (7 of 7) and 100% of terrace breccias sampled (7 of 7) yielded resolvable U-Th age estimates, while only one of four dome samples returned a reliable age. Reliable ages tended to be unresolvable on samples with very low ^{230}Th /^{232}Th ratios (e.g. ^{230}Th /^{232}Th <7) indicating a significant detrital component (S3 Table). It was not possible to resolve reliable or precise ages for any of the barrage samples, three dome and two cascade samples, however some of the corrected ages for these samples may provide a useful upper limit age estimate, i.e. the corrected age plus the associated 2σ uncertainty (S3 Table).

Table 1. U-Th age data for tufa samples from Ga-Mohana Hill.

Sample ID	Tufa type	^{238}U (ng/g)	$^{230}Th/^{238}U$	2σ	$^{234}U/^{238}U$	2σ	$^{230}Th/^{232}Th$	U-Th age (ka)	2σ	% error
18-10.2	dome	174	0.088	0.001	2.443	0.006	6.5	3.0	0.9	30
GHN-2	cascade	75	0.184	0.002	2.728	0.009	36.2	7.266	0.315	4.3
GHS-5	cascade	263	0.251	0.002	1.866	0.007	4.6	10.738	4.932	45.9
17-8.1	terrace	363	0.707	0.002	1.895	0.003	88.4	48.306	0.684	1.4
17-8.2	terrace	273	0.528	0.002	1.894	0.003	238.3	34.503	0.231	0.7
17-8.3	terrace	321	0.621	0.002	1.896	0.005	3367.7	41.760	0.220	0.5
17-8.4	terrace	298	0.641	0.003	1.903	0.005	4516.0	43.230	0.270	0.6
17-8.5	terrace	307	0.618	0.003	1.895	0.005	4858.7	41.620	0.260	0.6
17-8.6	terrace	459	0.503	0.004	1.899	0.006	216.6	32.450	0.370	1.1
GHN-1	rim pool	236	0.576	0.005	1.863	0.006	716.3	39.126	0.398	1.0
GHN-1.2	rim pool	234	0.543	0.005	1.859	0.006	6818.5	36.550	0.390	1.1
GHN-1.3	rim pool	240	0.551	0.005	1.868	0.007	10846.4	37.020	0.420	1.1
GHS-6	rim pool	212	0.864	0.007	1.914	0.007	41.3	60.379	1.809	3.0
GHS-6.1	rim pool	180	0.852	0.005	1.913	0.007	50.3	59.677	1.461	2.4
GHS-6.2	rim pool	190	0.873	0.003	1.928	0.0054	251.3	61.986	0.466	0.8
GHS-6.3	rim pool	158	0.798	0.003	1.882	0.004	15.9	53.100	4.200	7.9
18-7	terrace	847	0.749	0.003	1.833	0.005	50.5	53.520	1.310	2.4
18-13.1	cascade	249	1.174	0.003	2.654	0.007	68.3	58.610	0.990	1.7
18-13.2	cascade	226	1.313	0.003	2.742	0.007	93.6	65.040	0.810	1.2
18-13.3	cascade	132	1.287	0.004	2.646	0.007	620.5	67.150	0.380	0.6
18-13.4	cascade	195	1.483	0.004	2.933	0.008	124.6	69.830	0.680	1.0
18-14.1	cascade	83	1.308	0.009	2.644	0.009	243.4	68.430	0.730	1.1
18-14.2	cascade	139	1.219	0.006	2.551	0.008	46.1	64.280	1.600	2.5
18-14.3	cascade	98	1.351	0.008	2.705	0.009	439.1	69.350	0.670	1.0
18-14.4	cascade	180	1.481	0.006	2.876	0.008	47.0	70.600	1.670	2.4
18-15.1	cascade	137	1.319	0.007	2.668	0.008	781.0	68.520	0.570	0.8
18-15.2	cascade	95	1.317	0.011	2.587	0.010	746.9	71.340	0.890	1.2
18-15.3	cascade	313	1.522	0.005	2.940	0.008	229.7	72.280	0.530	0.7
18-17.1	cascade	154	2.176	0.006	3.194	0.006	29.0	102.900	3.200	3.1
18-17.2	cascade	148	2.085	0.007	3.102	0.006	44.2	102.100	2.100	2.1
18-17.3	cascade	142	2.217	0.006	3.289	0.006	95.7	103.310	1.080	1.0
18-16.1	cascade	164	2.586	0.008	3.614	0.007	32.9	110.600	3.000	2.7
18-16.2	cascade	177	2.404	0.007	3.476	0.007	43.9	105.900	2.200	2.1

The samples are labelled according to the sequence they were collected in but presented in stratigraphic order. Errors on all isotope activity ratios are reported with 2σ uncertainty. All ages have been corrected to account for the effect of detrital Th assuming an estimate for initial

$^{230}Th/^{232}Th$ of 1.5 ± 1.5, and calculated using the ^{230}Th-^{238}U decay constants of Cheng et al.[42] and equation 1 from Hellstrom[35].

The tufa ages span the last interglacial cycle, from 110.6 ± 3.0 ka through to 3.0 ± 0.9 ka (Table 1, Fig 3). The ages are clustered, suggesting episodic growth over this time, with at least four intervals of tufa formation at Ga-Mohana Hill identified at approximately 114-100 ka, 73-48 ka, 44-32 ka, and 15-2 ka (Fig 3). The 2σ uncertainties associated with the ages are small; most samples are associated with errors of <3 ka (on average approximately 1 ka) except for two samples, GHS-5 and GHS-6.3, which have an uncertainty of 4.9 ka (49%) and 4.2 ka (7.9%) respectively. These larger errors are due to a high detrital thorium component (Table 1).

Fig 3. Composite plot of Ga-Mohana Hill tufa formation compared to selected global proxies over the last 120 ka. (**A**) LR04 curve[46]; (**B**) variance of reconstructed sea surface temperatures (SST) from Indian Ocean core MD96-2048[47]; (**C**) mean daily summer insolation curve for 27°S[48]; (**D**) OSL age data from the Ga-Mohana Hill North excavation sediments[19, 24]; (**E**) tufa U-Th age data with 2σ error bars presented in Table 1.

The ages for the timing of human occupation at Ga-Mohana Hill coincides with three of the tufa forming intervals during MIS 5d, late MIS 3, and late MIS 2, indicating contemporaneous human activity and tufa precipitation at Ga-Mohana during those periods (Fig 3). The age certainty for the interval of tufa formation that overlaps with the MIS 2 occupation at Ga-Mohana Hill is less secure than the other intervals as it has a large error associated with it. The human occupation falls within the 2σ uncertainty of the tufa age.

Comparison to global records

We compare the timing of tufa formation at Ga-Mohana Hill with global palaeoclimate proxies to consider potential forcing factors (Fig 3). There is no clear glacial/interglacial partitioning of tufa formation episodes, as evidenced by comparing our data to the LR04 d^{18}O benthic stack[46] (Fig 3). This adds to growing evidence that the wet/dry, interglacial/glacial dichotomy through which much of southern African palaeoclimates has traditionally been viewed is overly simplistic[9, 49-51]. While tufas in the northern hemisphere are typically associated with interglacial climate conditions[52-55], our record suggests tufa formation was semi-continuous across MIS 4 and MIS 3; similarly anomalous tufa growth is reported from other sites locally[29] and globally[30, 56]. This suggests that tufa formation is neither restricted to interglacial periods, nor is it a simple product of changing global climate states.

The principal conditions required for tufa formation are sufficient effective precipitation to recharge the aquifers and $CaCO_3$ supersaturation of those waters[26, 28, 55]. Productive soil and vegetation cover is necessary to enhance the pCO_2 of the percolating waters, and moderate temperatures which balance productivity, moisture and evaporation, are important secondary requirements[26, 30]. Tufa formation is thus sensitive to multiple environmental parameters, but ultimately provides direct evidence of fresh water and associated productivity on the landscape. Our record indicates that these conditions were met during the time intervals presented here in the southern Kalahari over the last ~110 ka.

The limiting factor for tufa formation in semi-arid, low latitude regions is water availability[28, 57]. The spatial and temporal variability of rainfall in this southern Kalahari region is poorly constrained, but is thought to be modulated by summer insolation, with increased precipitation corresponding to insolation maxima[58]. However, there is no simple correlation between Ga-Mohana tufa formation and insolation. Based on the mean summer insolation curve for

27°S[48] (Fig 3), tufa formation during the 114-100 ka and 44-32 ka intervals coincide with increasing summer insolation, while tufa formation during 73-48 ka is variable, and at a minimum during the most recent 15-2 ka episode. It has been suggested that direct insolation forcing has played a lesser role over the last ~50 ka due to lower amplitude changes related to declining eccentricity[59], and that after ~70 ka, high latitude changes may have had a greater influence on southern African hydroclimate [60].

Following that warmer sea surface temperatures (SST) in the southwest Indian Ocean generate increased moisture and correlate to periods of greater rainfall in southeastern Africa[61-63], one might predict that past periods of warmer SST would correspond to periods of tufa formation at Ga-Mohana. However, tufa formation occurs across a range of Indian Ocean SST[47] (Fig 3) suggesting that SST is not the driving mechanism for increased rainfall in this region. While warmer SSTs coupled with a negative Southern Oscillation Index is suggested as contributing to higher rainfall during the 114-100 ka interval[19], it is likely that the primary driving mechanism for rainfall in this region has varied over time[59].

Comparison to regional records

We compare the record of tufa formation intervals at Ga-Mohana Hill with other palaeoenvironmental records at nearby Kathu Pan and Wonderwerk Cave (Fig 4). These three sites all occur within ~60 km of each other and are likely to have experienced the same climate systems. At Kathu Pan, sediment analysis indicates wet conditions through much of MIS 5 and 4, consistent with the tufa record at Ga-Mohana Hill. Marshy conditions prevailed at Kathu Pan from ~101-80 ka, and palygorskite-coated sands indicate the presence of fluctuating water levels across five intervals between ~167-52 ka[17]. By ~23 ka, the sedimentary record shifts to one characterized by extensive pedogenic carbonate deposits that indicate drier conditions, and perhaps more seasonal rainfall

compared to earlier time periods[17]. This indication for drier conditions at Kathu Pan during the Last Glacial Maximum (LGM) is consistent with the lack of evidence for tufa formation during that time at Ga-Mohana Hill. However, Ga-Mohana Hill documents a subsequent late glacial wet period commencing as early as ~15 ka. At Wonderwerk Cave, a combined speleothem isotope and pollen record indicates a climate fluctuating between wet and dry conditions through the LGM and late glacial[64]. Wetter conditions are reflected in the pollen and stable isotope record at ~35-33 ka, from 23 to 17 ka, and from 4 ka to present[64]. While the earlier and later parts of this sequence are consistent with the records at Ga-Mohana Hill and Kathu Pan, evidence for wetter conditions during the LGM is inconsistent.

Fig 4. Comparison of palaeoenvironmental and archaeological records from Ga-Mohana Hill, Kathu Pan, and Wonderwerk Cave. Archaeological occupation ages for Ga-Mohana Hill[19, 24], Kathu Pan[17, 18, 65, 66] and Wonderwerk Cave[13, 67, 68]. Pale orange bars highlight periods of occupation. Palaeoenvironment proxy data from Ga-Mohana Hill (this study), Kathu Pan[17] and Wonderwerk Cave[13, 64]. Pale blue bars highlight wet periods across the sites.

The proximity of Ga-Mohana Hill, Kathu Pan, and Wonderwerk Cave to each other means that they are likely to have been utilized by the same groups of mobile hunter-gatherers, thus providing an opportunity to consider the relationships between wet periods and evidence for human occupation in this region of the southern Kalahari (Fig 4). Between ~251 and 138 ka at Wonderwerk Cave, there is evidence for both wetter conditions and human occupation.[13] Archaeological material at Kathu Pan occurs within palygorskite-coated, water-associated sediments dated to ~156 ka, ~121 ka and ~74 ka[17], with the latter being a Howiesons Poort occurrence. Also at Kathu Pan, wet, marshy conditions that are likely to have supported a significant amount of vegetation occur between ~101 and 80 ka, coupled with evidence for human occupation[17]. At Ga-Mohana Hill, tufa

formation at ~114-100 ka correlates with human occupation at the site. Thus, in summary, before ~71 ka, human occupation of the region appears to have been associated with the availability of water.

After ~71 ka, the timing of human occupation and wet periods are decoupled (Fig 4). Tufas at Ga-Mohana Hill indicate that much of MIS 4 and 3 is characterised by wet conditions. The sediments at Kathu Pan continue to indicate the presence of water through much of MIS 4, although the organic-rich marsh sediments do not occur after MIS 5[17]. However, evidence for human occupation during this time is lacking at both Kathu Pan and Ga-Mohana Hill[24]. There are Middle Stone Age deposits at Wonderwerk Cave that have not yet been securely dated that could potentially represent this period[8], but this remains unknown at this point. At ~35-31 ka, wet conditions are represented at Ga-Mohana Hill and Wonderwerk Cave[64], with human occupation evidenced at Ga-Mohana Hill[24] and Kathu Pan[66]. Human occupation is evident at Ga-Mohana Hill[24] during the late glacial, associated with evidence for relatively wetter conditions, and at Kathu Pan[65, 66] during the LGM associated with evidence for relatively drier conditions. Late glacial deposits at Wonderwerk Cave indicate an association of dry conditions and human occupation[68]. Thus, MIS 2 provides very little coherence with respect to the relationship between water availability and human occupation. There is persistent evidence for human occupation through the Holocene despite changes in palaeoenvironmental conditions[17, 66, 68].

Discussion

In this semi-arid region with limited, seasonal rainfall and no evidence of actively precipitating tufa, the relict tufa deposits at Ga-Mohana Hill are a record of past periods of increased water on the landscape, and climatic conditions favourable for tufa formation. In summary, we show that periods of tufa formation were punctuated over the last 110 ka, and that U-Th dating of the tufas,

buoyed by the laser ablation pre-screening method employed here, provides a valuable tool for investigating past environmental change in this region of the southern Kalahari.

Wet periods in the southern Kalahari were not restricted to interglacials. Ga-Mohana Hill shows extensive tufa formation during much of MIS 4, a period generally assumed to be characterised by typical cold and dry glacial conditions across much of the interior of southern Africa[69]. Increased water availability during this time is supported by other palaeoenvironmental records of the Kalahari Basin, such as at Kathu Pan discussed above. Furthermore, at Witpan Dunes, approximately 350 km to the north west, the absence of southern Kalahari dune data during MIS 4[70] indicates unfavourable conditions for dune accumulation, suggesting increased rainfall, decreased windiness, and a denser vegetation cover[71]. To the north, a Makgadikgadi Megalake highstand has been dated to 64.2 ± 2.0 ka suggesting there was also substantial water availability in the Middle Kalahari at that time[72]. Comparisons with global paleoenvironmental proxies indicate that no single factor explains the timing of the past wet, tufa-forming periods, and that hydrological dynamics in the southern Kalahari were influenced by multiple factors operating at various scales. The tufa intervals represent a southern Kalahari environment characterised by a positive hydrological balance and mild temperatures favourable for productive vegetation and soils. Our results challenge global generalisations of past climate change, in accordance with other studies that highlight the necessity for regionally specific models[10, 73, 74].

In the southern Kalahari, early human population distributions appear to have been modulated by water availability before ~71 ka but not after. Despite evidence for wetter conditions, archaeological deposits dating to MIS 4 and the early part of MIS 3 have not yet been identified in the punctuated record of human occupation at Ga-Mohana Hill, nor at nearby Kathu Pan or Wonderwerk Cave. Future work is required to determine whether this absence of evidence is

evidence of absence, or whether issues with site formation, site visibility, and/or dating challenges explain why no archaeological deposits have yet been identified. This work will be key for further testing hypotheses that link early human population distribution patterns to water availability, potential refugia conditions, and interglacial/glacial cycling[4-8].

The time interval corresponding to MIS 2 provides little coherence with respect to the relationship between water availability and human occupation. The three records considered here do not agree on whether conditions were wetter or drier during the LGM and humans appeared to have occupied the region through both the LGM and late glacial. Others have highlighted that the palaeoenvironmental record for MIS 2 across the Kalahari Basin and surrounding regions is complex, documenting a high degree of spatial and temporal variability[10]. This lack of coherence may be in part due to the variable responses of palaeoenvironmental proxies to temperature and water availability changes, and potentially lags in responses. A shift in seasonality may also play a role, with some proxies responding to seasonality changes for precipitation, as evidenced at Kathu Pan[17], as opposed to mean annual precipitation. The higher frequency of well-preserved and datable MIS 2 palaeoenvironmental and archaeological records in the Kalahari Basin compared to earlier glacial periods[20] means there is ample opportunity to further explore human-environment interaction during the LGM and late glacial.

Conclusion

Identifying the timing and nature of human occupation in the Kalahari Desert is critical for understanding the emergence of our ability to adapt to new and extreme environments[2]. For a long time, the Kalahari Desert has been considered too arid for early human populations to persist, and evidence for occupation was assumed to represent wetter periods. Until now, a rarity of integrated

palaeoenvironmental and archaeological records has largely prevented adequate testing of these assumptions. The results presented here challenge the traditional view, showing that by approximately 71 ka, water availability alone did not mediate Late Pleistocene human occupation in the southern Kalahari Desert. This decoupling of human occupation and wet phases could reflect new social and technological adaptations that helped hunter-gatherers cope more effectively with diverse environmental conditions.

Acknowledgements

Thank you to the Baga Motlhware Traditional Council and South African Heritage Resources Agency for permissions to work at Ga-Mohana Hill. We acknowledge Serene Paul, Roland Maas, Bence Paul and Russell Drysdale at the University of Melbourne for assistance with the U-Th analyses and laser ablation, and Renee Van Der Merwe at the University of Cape Town for preparing thin sections. We also acknowledge David Morris and the McGregor Museum, Simon Hall, Andy Herries, Kyle Brown and Sechaba Maape.

References

1. Deacon HJ. Demography, subsistence, and culture during the Acheulian in southern Africa. After the australopithecines: De Gruyter Mouton; 2011. p. 543-70.
2. Roberts P, Stewart BA. Defining the 'generalist specialist'niche for Pleistocene Homo sapiens. Nature Human Behaviour. 2018;2(8):542-50.
3. Sampson CG, Moore V, Bousman CB, Stafford B, Giordano A, Willis M. A GIS analysis of the Zeekoe Valley Stone Age archaeological record in South Africa. Journal of African Archaeology. 2015;13(2):167-85.
4. Butzer KW. Geo-archeological interpretation of Acheulian calc-pan sites at Doornlaagte and Rooidam (Kimberley, South Africa). Journal of Archaeological Science. 1974;1(1):1-25.
5. Mirazón Lahr M, Foley RA. Towards a theory of modern human origins: geography, demography, and diversity in recent human evolution. American Journal of Physical Anthropology: The Official Publication of the American Association of Physical Anthropologists. 1998;107(S27):137-76.
6. Marean CW. Pinnacle Point Cave 13B (Western Cape Province, South Africa) in context: The Cape Floral kingdom, shellfish, and modern human origins. J Hum Evol. 2010;59(3-4):425-43. Epub 2010/10/12. doi: 10.1016/j.jhevol.2010.07.011. PubMed PMID: 20934095.

7. Beaumont PB, Bednarik RG. Concerning a cupule sequence on the edge of the Kalahari Desert in South Africa. Rock Art Research. 2015;32(2):163.
8. Beaumont PB, Vogel JC. On a timescale for the past million years of human history in central South Africa. 2006.
9. Burrough SL. Late Quaternary environmental change and human occupation of the southern African interior. Africa from MIS 6-2: Springer; 2016. p. 161-74.
10. Lukich V, Ecker M. Pleistocene environments in the southern Kalahari of South Africa. Quaternary International. 2021. doi: 10.1016/j.quaint.2021.03.008.
11. Bamford MK. Macrobotanical Remains from Wonderwerk Cave (Excavation 1), Oldowan to Late Pleistocene (2 Ma to 14 ka bp), South Africa. African Archaeological Review. 2015;32(4):813-38. doi: 10.1007/s10437-015-9200-0.
12. Brook GA, Railsback LB, Scott L, Voarintsoa NRG, Liang F. Late Holocene Stalagmite and Tufa Climate Records for Wonderwerk Cave: Relationships Between Archaeology and Climate in Southern Africa. African Archaeological Review. 2015;32(4):669-700. doi: 10.1007/s10437-015-9210-y.
13. Chazan M, Berna F, Brink J, Ecker M, Holt S, Porat N, et al. Archeology, Environment, and Chronology of the Early Middle Stone Age Component of Wonderwerk Cave. Journal of Paleolithic Archaeology. 2020;3(3):302-35. doi: 10.1007/s41982-020-00051-8.
14. Ecker M, Brink J, Horwitz LK, Scott L, Lee-Thorp JA. A 12,000 year record of changes in herbivore niche separation and palaeoclimate (Wonderwerk Cave, South Africa). Quaternary Science Reviews. 2018;180:132-44. doi: 10.1016/j.quascirev.2017.11.025.
15. Lee-Thorp JA, Ecker M. Holocene environmental change at Wonderwerk Cave, South Africa: Insights from stable light isotopes in ostrich eggshell. African Archaeological Review. 2015;32(4):793-811.
16. Rossouw L. An early Pleistocene phytolith record from Wonderwerk cave, northern Cape, South Africa. African Archaeological Review. 2016;33(3):251-63.
17. Lukich V, Cowling S, Chazan M. Palaeoenvironmental reconstruction of Kathu Pan, South Africa, based on sedimentological data. Quaternary Science Reviews. 2020;230. doi: 10.1016/j.quascirev.2019.106153.
18. Lukich V, Porat N, Faershtein G, Cowling S, Chazan M. New Chronology and Stratigraphy for Kathu Pan 6, South Africa. Journal of Paleolithic Archaeology. 2019;2(3):235-57. doi: 10.1007/s41982-019-00031-7.
19. Wilkins J, Schoville BJ, Pickering R, Gliganic L, Collins B, Brown KS, et al. Innovative Homo sapiens behaviours 105,000 years ago in a wetter Kalahari. Nature. 2021;592(7853):248-52. Epub 2021/04/02. doi: 10.1038/s41586-021-03419-0. PubMed PMID: 33790469.
20. Wilkins J. Homo sapiens origins and evolution in the Kalahari Basin, southern Africa. Evolutionary Anthropology: Issues, News, and Reviews. 2021.
21. Schoville BJ, Brown KS, Wilkins J. A Lithic Provisioning Model as a Proxy for Landscape Mobility in the Southern and Middle Kalahari. Journal of Archaeological Method and Theory. 2021:1-26.
22. Beukes NJ. Facies relations, depositional environments and diagenesis in a major early Proterozoic stromatolitic carbonate platform to basinal sequence, Campbellrand Subgroup, Transvaal Supergroup, Southern Africa. Sedimentary Geology. 1987;54(1-2):1-46.
23. Scott L, Thackeray JF. Palynology of Holocene Deposits in Excavation 1 at Wonderwerk Cave, Northern Cape (South Africa). African Archaeological Review. 2015;32(4):839-55. doi: 10.1007/s10437-015-9204-9.
24. Wilkins J, Schoville BJ, Brown KS, Gliganic L, Meyer MC, Loftus E, et al. Fabric Analysis and Chronology at Ga-Mohana Hill North Rockshelter, Southern Kalahari Basin: Evidence for In Situ, Stratified Middle and Later Stone Age Deposits. Journal of Paleolithic Archaeology. 2020;3(3):336-61. doi: 10.1007/s41982-020-00050-9.

25. Ford T, Pedley H. A review of tufa and travertine deposits of the world. Earth-Science Reviews. 1996;41(3-4):117-75.

26. Pentecost A. Travertine: Springer Science & Business Media; 2005.

27. Andrews JE. Palaeoclimatic records from stable isotopes in riverine tufas: Synthesis and review. Earth-Science Reviews. 2006;75(1-4):85-104. doi: 10.1016/j.earscirev.2005.08.002.

28. Cremaschi M, Zerboni A, Spötl C, Felletti F. The calcareous tufa in the Tadrart Acacus Mt. (SW Fezzan, Libya). Palaeogeography, Palaeoclimatology, Palaeoecology. 2010;287(1-4):81-94. doi: 10.1016/j.palaeo.2010.01.019.

29. Doran TL, Herries AI, Hopley PJ, Sombroek H, Hellstrom J, Hodge E, et al. Assessing the paleoenvironmental potential of Pliocene to Holocene tufa deposits along the Ghaap Plateau escarpment (South Africa) using stable isotopes. Quaternary Research. 2015;84(1):133-43.

30. Sancho C, Arenas C, Vázquez-Urbez M, Pardo G, Lozano MV, Peña-Monné JL, et al. Climatic implications of the Quaternary fluvial tufa record in the NE Iberian Peninsula over the last 500 ka. Quaternary Research. 2015;84(3):398-414. doi: 10.1016/j.yqres.2015.08.003.

31. Garnett ER, Gilmour MA, Rowe PJ, Andrews JE, Preece RC. 230Th/234U dating of Holocene tufas: possibilities and problems. Quaternary Science Reviews. 2004;23(7-8):947-58. doi: 10.1016/j.quascirev.2003.06.018.

32. Stone AEC, Viles HA, Thomas L, Van Calsteren P. Can 234U-230Th dating be used to date large semi-arid tufas? Challenges from a study in the Naukluft Mountains, Namibia. Journal of Quaternary Science. 2010;25(8):1360-72. doi: 10.1002/jqs.1435.

33. Pickering R, Hancox PJ, Lee-Thorp JA, Grun R, Mortimer GE, McCulloch M, et al. Stratigraphy, U-Th chronology, and paleoenvironments at Gladysvale Cave: insights into the climatic control of South African hominin-bearing cave deposits. J Hum Evol. 2007;53(5):602-19. Epub 2007/10/09. doi: 10.1016/j.jhevol.2007.02.005. PubMed PMID: 17920104.

34. Maape S. Drawing creepy places: Representing liminal ritual spaces of Kuruman, South Africa. Dialectic IX. in press.

35. Hellstrom J. U–Th dating of speleothems with high initial 230Th using stratigraphical constraint. Quaternary Geochronology. 2006;1(4):289-95. doi: 10.1016/j.quageo.2007.01.004.

36. Woodhead JD, Hellstrom J, Hergt JM, Greig A, Maas R. Isotopic and elemental imaging of geological materials by laser ablation inductively coupled plasma-mass spectrometry. Geostandards and Geoanalytical Research. 2007;31(4):331-43.

37. Paton C, Hellstrom J, Paul B, Woodhead J, Hergt J. Iolite: Freeware for the visualisation and processing of mass spectrometric data. Journal of Analytical Atomic Spectrometry. 2011;26(12). doi: 10.1039/c1ja10172b.

38. Paul B, Paton C, Norris A, Woodhead J, Hellstrom J, Hergt J, et al. CellSpace: A module for creating spatially registered laser ablation images within the Iolite freeware environment. Journal of Analytical Atomic Spectrometry. 2012;27(4). doi: 10.1039/c2ja10383d.

39. Green H, Gleadow A, Finch D. Characterisation of mineral deposition systems associated with rock art in the Kimberley region of northwest Australia. Data Brief. 2017;14:813-35. Epub 2017/10/13. doi: 10.1016/j.dib.2017.08.029. PubMed PMID: 29021996; PubMed Central PMCID: PMCPMC5633166.

40. Hellstrom J. Rapid and accurate U/Th dating using parallel ion-counting multi-collector ICP-MS. Journal of Analytical Atomic Spectrometry. 2003;18(11). doi: 10.1039/b308781f.

41. Drysdale RN, Paul BT, Hellstrom JC, Couchoud I, Greig A, Bajo P, et al. Precise microsampling of poorly laminated speleothems for U-series dating. Quaternary Geochronology. 2012;14:38-47. doi: 10.1016/j.quageo.2012.06.009.

42. Cheng H, Lawrence Edwards R, Shen C-C, Polyak VJ, Asmerom Y, Woodhead J, et al. Improvements in 230Th dating, 230Th and 234U half-life values, and U–Th isotopic measurements by multi-collector inductively coupled plasma mass spectrometry. Earth and Planetary Science Letters. 2013;371-372:82-91. doi: 10.1016/j.epsl.2013.04.006.

43. Altermann W, Wotherspoon JM. The carbonates of the Transvaal and Griqualand West sequences of the Kaapvaal craton, with special reference to the Lime Acres limestone deposit. Mineralium Deposita. 1995;30(2). doi: 10.1007/bf00189341.
44. Smit P. Groundwater recharge in the dolomite of the Ghaap Plateau near Kuruman in the Northern Cape, Republic of South Africa. Water SA. 1978;4(2):81-92.
45. Manzo E, Perri E, Tucker ME. Carbonate deposition in a fluvial tufa system: processes and products (Corvino Valley - southern Italy). Sedimentology. 2012;59(2):553-77. doi: 10.1111/j.1365-3091.2011.01266.x.
46. Lisiecki LE, Raymo ME. A Pliocene-Pleistocene stack of 57 globally distributed benthic δ18O records. Paleoceanography. 2005;20(1):n/a-n/a. doi: 10.1029/2004pa001071.
47. Caley T, Extier T, Collins JA, Schefuss E, Dupont L, Malaize B, et al. A two-million-year-long hydroclimatic context for hominin evolution in southeastern Africa. Nature. 2018;560(7716):76-9. Epub 2018/07/11. doi: 10.1038/s41586-018-0309-6. PubMed PMID: 29988081.
48. Laskar J, Robutel P, Joutel F, Gastineau M, Correia A, Levrard B. A long-term numerical solution for the insolation quantities of the Earth. Astronomy & Astrophysics. 2004;428(1):261-85.
49. Collins JA, Schefuß E, Govin A, Mulitza S, Tiedemann R. Insolation and glacial–interglacial control on southwestern African hydroclimate over the past 140 000 years. Earth and Planetary Science Letters. 2014;398:1-10.
50. Blome MW, Cohen AS, Tryon CA, Brooks AS, Russell J. The environmental context for the origins of modern human diversity: a synthesis of regional variability in African climate 150,000-30,000 years ago. J Hum Evol. 2012;62(5):563-92. Epub 2012/04/20. doi: 10.1016/j.jhevol.2012.01.011. PubMed PMID: 22513381.
51. Chase BM. South African palaeoenvironments during marine oxygen isotope stage 4: a context for the Howiesons Poort and Still Bay industries. Journal of Archaeological Science. 2010;37(6):1359-66. doi: 10.1016/j.jas.2009.12.040.
52. Soligo M, Tuccimei P, Barberi R, Delitala M, Miccadei E, Taddeucci A. U/Th dating of freshwater travertine from Middle Velino Valley (Central Italy): paleoclimatic and geological implications. Palaeogeography, Palaeoclimatology, Palaeoecology. 2002;184(1-2):147-61.
53. Ordóñez S, González Martín JA, García del Cura MA, Pedley HM. Temperate and semi-arid tufas in the Pleistocene to Recent fluvial barrage system in the Mediterranean area: The Ruidera Lakes Natural Park (Central Spain). Geomorphology. 2005;69(1-4):332-50. doi: 10.1016/j.geomorph.2005.02.002.
54. Martín-Algarra An, Martín-Martín M, Andreo B, Julià R, González-Gómez C. Sedimentary patterns in perched spring travertines near Granada (Spain) as indicators of the paleohydrological and paleoclimatological evolution of a karst massif. Sedimentary Geology. 2003;161(3-4):217-28. doi: 10.1016/s0037-0738(03)00115-5.
55. Domínguez-Villar D, Vázquez-Navarro JA, Cheng H, Edwards RL. Freshwater tufa record from Spain supports evidence for the past interglacial being wetter than the Holocene in the Mediterranean region. Global and Planetary Change. 2011;77(3-4):129-41. doi: 10.1016/j.gloplacha.2011.04.006.
56. Auler AS, Smart PL. Late Quaternary paleoclimate in semiarid northeastern Brazil from U-series dating of travertine and water-table speleothems. Quaternary Research. 2001;55(2):159-67.
57. Viles HA, Taylor MP, Nicoll K, Neumann S. Facies evidence of hydroclimatic regime shifts in tufa depositional sequences from the arid Naukluft Mountains, Namibia. Sedimentary Geology. 2007;195(1-2):39-53. doi: 10.1016/j.sedgeo.2006.07.007.
58. Partridge TC. Cainozoic environmental change in southern Africa, with special emphasis on the last 200 000 years. Progress in Physical Geography. 1997;21(1):3-22.

59. Singarayer JS, Burrough SL. Interhemispheric dynamics of the African rainbelt during the late Quaternary. Quaternary Science Reviews. 2015;124:48-67. doi: 10.1016/j.quascirev.2015.06.021.
60. Chase B, Harris C, de Wit MJ, Kramers J, Doel S, Stankiewicz J. South African speleothems reveal influence of high- and lowlatitude
forcing over the past 113.5 k.y. Geology. 2021. doi: 10.1130/g49323.1.
61. Reason CJC. Subtropical Indian Ocean SST dipole events and southern African rainfall. Geophysical Research Letters. 2001;28(11):2225-7. doi: 10.1029/2000gl012735.
62. Reason CJC. Sensitivity of the southern African circulation to dipole sea-surface temperature patterns in the south Indian Ocean. International Journal of Climatology. 2002;22(4):377-93. doi: 10.1002/joc.744.
63. Chevalier M, Chase BM. Southeast African records reveal a coherent shift from high- to low-latitude forcing mechanisms along the east African margin across last glacial–interglacial transition. Quaternary Science Reviews. 2015;125:117-30. doi: 10.1016/j.quascirev.2015.07.009.
64. Brook GA, Scott L, Railsback LB, Goddard EA. A 35ka pollen and isotope record of environmental change along the southern margin of the Kalahari from a stalagmite and animal dung deposits in Wonderwerk Cave, South Africa. Journal of Arid Environments. 2010;74(7):870-84. doi: 10.1016/j.jaridenv.2009.11.006.
65. Porat N, Chazan M, Grün R, Aubert M, Eisenmann V, Horwitz LK. New radiometric ages for the Fauresmith industry from Kathu Pan, southern Africa: Implications for the Earlier to Middle Stone Age transition. Journal of Archaeological Science. 2010;37(2):269-83. doi: 10.1016/j.jas.2009.09.038.
66. Beaumont PB, Morris D. Guide to Archaeological Sites in the Northern Cape: Prepared for the Southern African Association of Archaeologists Post-conference Excursion, 9th-13th September 1990: McGregor Museum; 1990.
67. Chazan M, Avery DM, Bamford MK, Berna F, Brink J, Fernandez-Jalvo Y, et al. The Oldowan horizon in Wonderwerk Cave (South Africa): archaeological, geological, paleontological and paleoclimatic evidence. J Hum Evol. 2012;63(6):859-66. Epub 2012/11/07. doi: 10.1016/j.jhevol.2012.08.008. PubMed PMID: 23127763.
68. Ecker M, Brink J, Chazan M, Horwitz LK, Lee-Thorp JA. Radiocarbon dates constrain the timing of environmental and cultural shifts in the Holocene strata of Wonderwerk Cave, South Africa. Radiocarbon. 2017;59(4):1067-86.
69. van Zinderen Bakker Sr E. The Late Quaternary history of climate and vegetation in East and southern Africa. Bothalia. 1983;14(3/4):369-75.
70. Telfer MW, Thomas DSG. Late Quaternary linear dune accumulation and chronostratigraphy of the southwestern Kalahari: implications for aeolian palaeoclimatic reconstructions and predictions of future dynamics. Quaternary Science Reviews. 2007;26(19-21):2617-30. doi: 10.1016/j.quascirev.2007.07.006.
71. Hesse PP, Magee JW, van der Kaars S. Late Quaternary climates of the Australian arid zone: a review. Quaternary International. 2004;118-119:87-102. doi: 10.1016/s1040-6182(03)00132-0.
72. Burrough SL, Thomas DSG, Bailey RM. Mega-Lake in the Kalahari: A Late Pleistocene record of the Palaeolake Makgadikgadi system. Quaternary Science Reviews. 2009;28(15-16):1392-411. doi: 10.1016/j.quascirev.2009.02.007.
73. Ecker M, Brink JS, Rossouw L, Chazan M, Horwitz LK, Lee-Thorp JA. The palaeoecological context of the Oldowan–Acheulean in southern Africa. Nature ecology & evolution. 2018;2(7):1080-6.
74. Thomas DSG, Burrough SL. Interpreting geoproxies of late Quaternary climate change in African drylands: Implications for understanding environmental change and early human behaviour. Quaternary International. 2012;253:5-17. doi: 10.1016/j.quaint.2010.11.001.

Supporting information

S1 Table. Tufa sample inventory. Samples labelled and listed in order of collection. Samples with GH prefix collected in 2016, numerical prefix of other samples indicates the year they were collected (e.g. 17- = 2017).

S1 Fig. Photographs of cascade hand and drill core samples. a,b) field context of sample 18-4; c) hand sample scan of 18-4 showing fine, undulating layers; d,e) field context of drill core samples 18-16 (g) and 18-17 (f); h) photograph of in-situ cascade tufa sampled with core drill; i) drill core sample 18-12; j,k) thin section photographs of sample 18-12 (in ppl) showing irregular, domal micritic laminae with lenses of microspar and micropores.

S2 Fig. Field photographs, hand samples and thin section photomicrographs of rim pool samples. a) Field context of sample 17-16 showing circular configuration and surface desiccation cracks; b,c) hand sample photographs showing 4cm layer of carbonate and cemented clasts on the underside; d) field context of sample GHN-1; e) hand sample scan of GHN-1; c) photograph of thin section from GHN-1 showing aphanitic fabric of biomicrite with spar-filled filamentous cavities.

S3 Fig. Photographs of terrace breccia hand samples. a) terrace breccia sample showing included detrital clasts and brecciated tufa clasts; b) hand sample scan of sample 18-7; c) field context and d) hand sample scan of sample 17-8 showing massive, dense micrite; e,f) thin section photographs of terrace sample 17-8 showing clotted fabric of peloidal micrite with microspar-filled void spaces.

S4 Fig. Photographs, hand sample scans and thin section photographs of barrage tufas. a) field

photograph of sample 17-6; b) hand sample scan of sample 17-6 showing irregular, undulating and discontinuous layering; c) thin section photographs show stromatolite-type micrite crustal laminae alternating with chaotic microspar laminae with detrital and oxide inclusions (c) and discontinuous crinkly microspar laminae with overprinting of oxide precipitates (f).

S5 Fig. Photographs of sampled domes. a) field context of tufa dome, sampled using an angle grinder; b) dense mm-scale layers alternating with irregular, porous and friable layers; c) photomicrograph of thin section from sample in (b) showing micro laminae; d,e) dome sampled with drill-core; f) hand sample of dome core showing large cavities and porous, reticulate framework.

S6 Fig. High resolution images of cascade (GHN2 and GHS5), terrace (17-8) and rim pool (GHN1, 18-7, GHS6) tufa samples overlain by LA-ICP-MS ^{238}U (left) and ^{232}Th (right) element distribution maps. Concentrations in ppm shown in adjacent colour scales (warmer colour = higher concentration). Black circles represent approximate locations of subsamples drilled for U-Th dating prior to pre-screening, and oblong free-forms show exact locations at which subsamples were drilled for U-Th dating following LA-ICP-MS analysis. Ages associated with each subsample are given in thousands of years (ka) and are reported in Table 1.

S7 Fig. High resolution images of cascade core samples (18-13, 18-14, 18-15, 18-16, 18-17) overlain by LA-ICP-MS ^{238}U (left) and ^{232}Th (right) element distribution maps. Concentrations shown in ppm in adjacent colour scales (warmer colour = higher concentration). Black circles represent approximate locations of subsamples drilled for U-Th dating prior to pre-screening, and oblong free-forms show exact locations at which subsamples were drilled for U-Th dating following LA-ICP-MS analysis. Ages associated with each subsample are given in thousands of years (ka) and are reported in Table 1.

S8 Fig. High resolution images of samples with unreliable and imprecise age solutions (Supplementary Table 3) with the exception of sample 18-10.2 (bottom of sample) which has an age of 3.0 ± 0.9 ka. Black oblong outline represents material drilled for U-Th dating. Cascade samples (18-4 and 18-12), barrage samples (17-6 and 18-6) and dome core samples (18-10) overlain by LA-ICPMS ^{238}U (left) and ^{232}Th (right) element distribution maps. Concentrations in ppm shown in adjacent colour scales (warmer colour = higher concentration).

S2 Table. U and Th isotope ratios measured in tufa samples with reliable and precise ages. The samples are labelled according to the sequence they were collected in but presented in stratigraphic order. Errors on all isotope activity ratios are reported with 2σ uncertainty. All ages have been corrected to account for the effect of detrital Th assuming an estimate for initial $^{230}Th/^{232}Th$ of 1.5 ± 1.5, and calculated using the ^{230}Th-^{238}U decay constants of Cheng et al.(73) and equation 1 from Hellstrom(66).

S3 Table. U and Th isotope ratios measured in tufa samples which have unreliable or imprecise age solutions. Errors on all isotope activity ratios are reported with 2σ uncertainty. Upper limit is defined as corrected age plus 2σ uncertainty. All ages have been corrected to account for the effect of detrital Th assuming an estimate for initial $^{230}Th/^{232}Th$ of 1.5 ± 1.5, and calculated using the ^{230}Th-^{238}U decay constants of Cheng et al.(73) and equation 1 from Hellstrom(66).

Figure 1

Click here to access/download;Figure;Fig1.tif

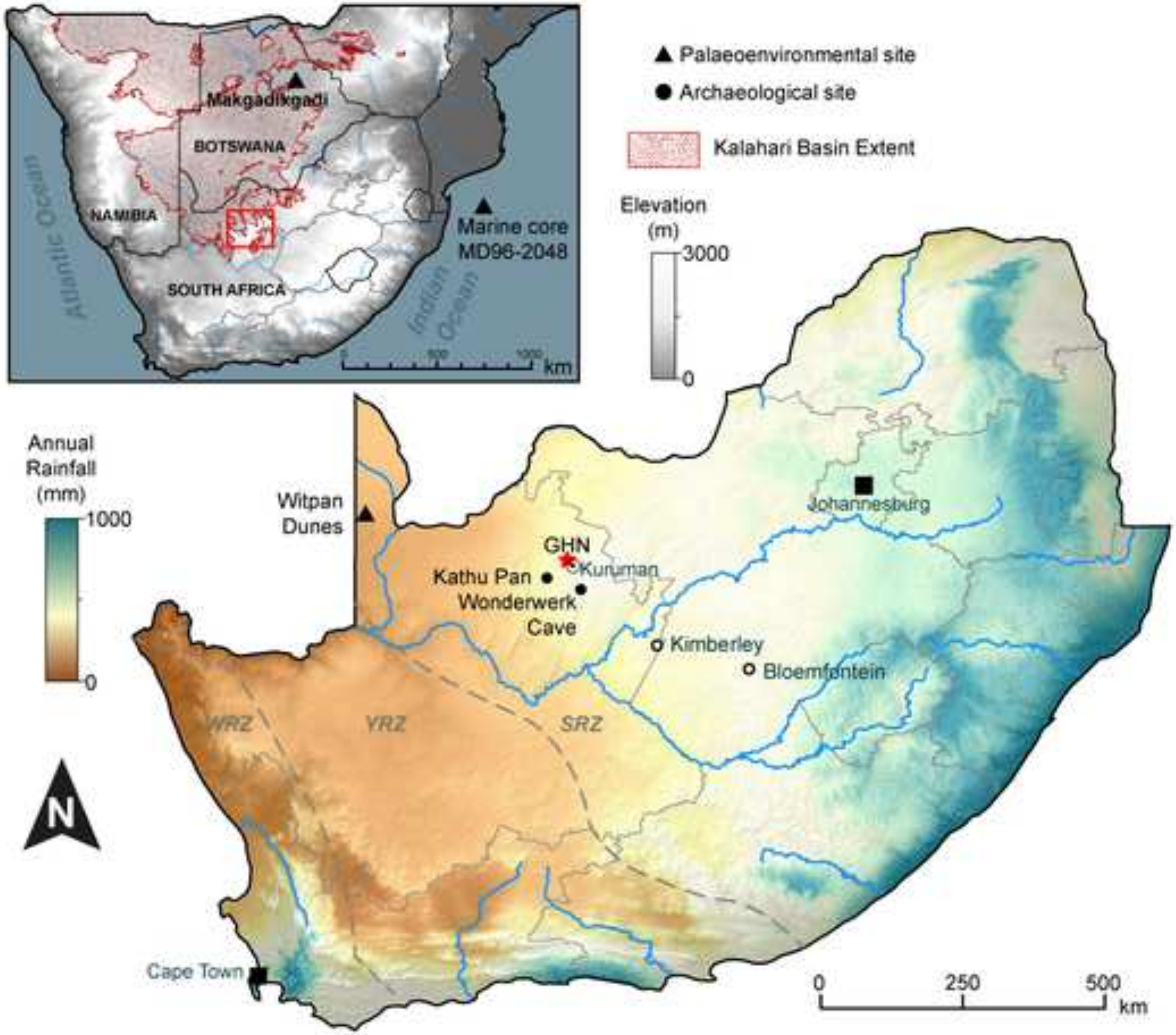

Figure 2

Click here to access/download;Figure;Fig2.tiff

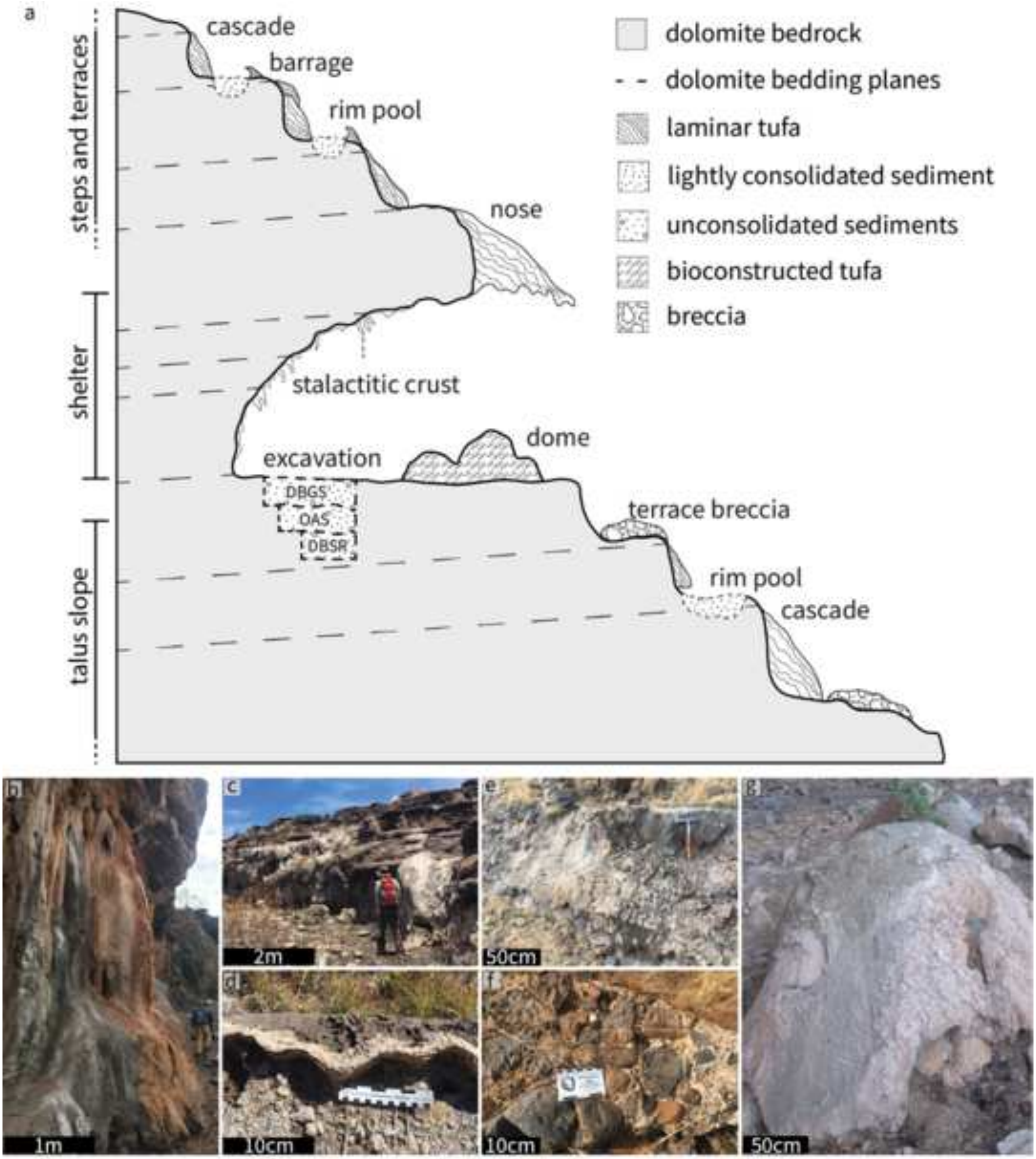

Figure 3

Click here to access/download;Figure;Fig3.tif

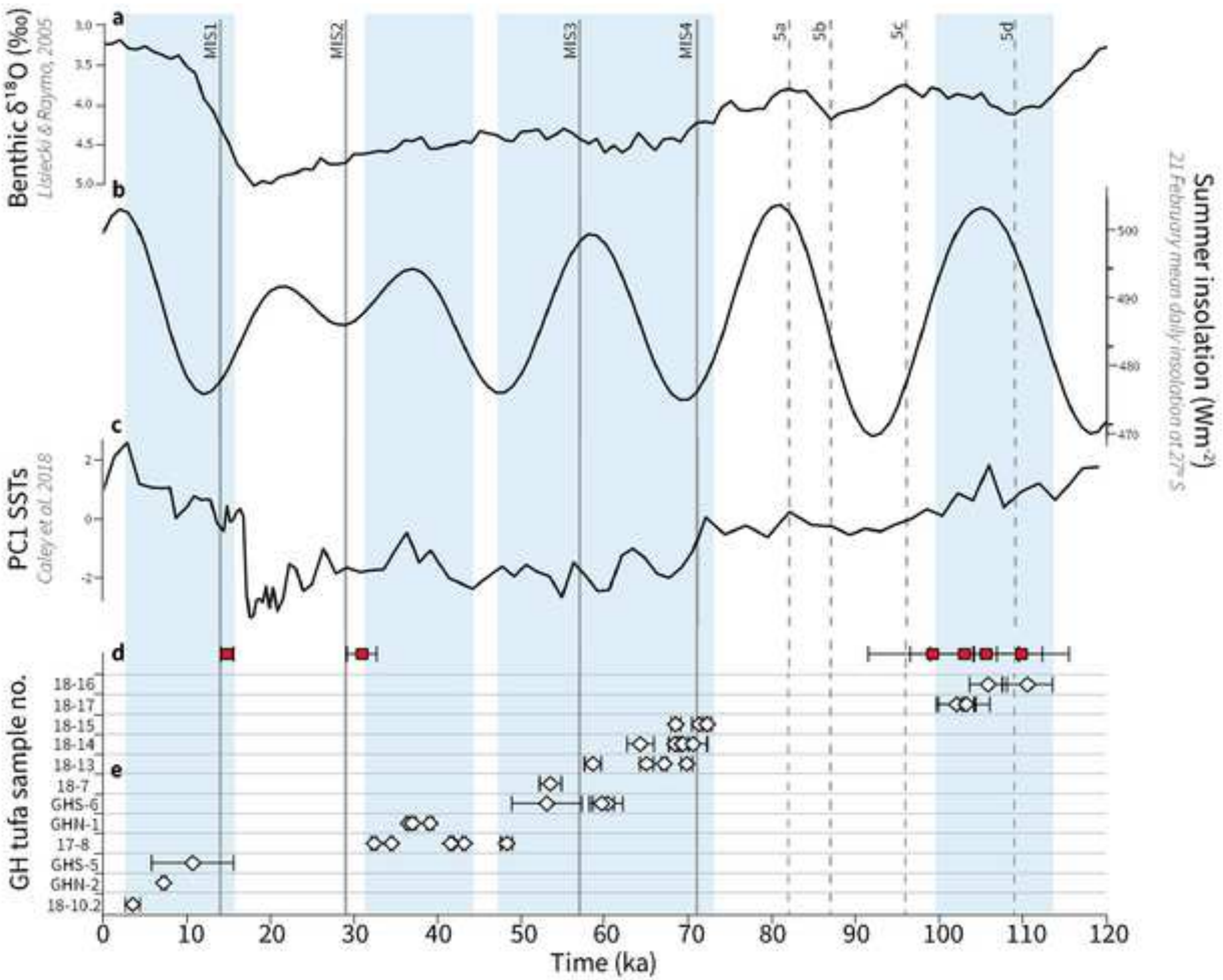

Episodes of tufa formation at Ga-Mohana Hill

Marine Isotope Stage and sub-stage boundaries (Lisiecki & Raymo, 2005)

Ga-Mohana Hill tufa U-Th age data (this study)

Ga-Mohana Hill archaeological occupation OSL ages (Wilkins et al. 2020; 2021)

Figure 4

Click here to access/download;Figure;Fig4.tif

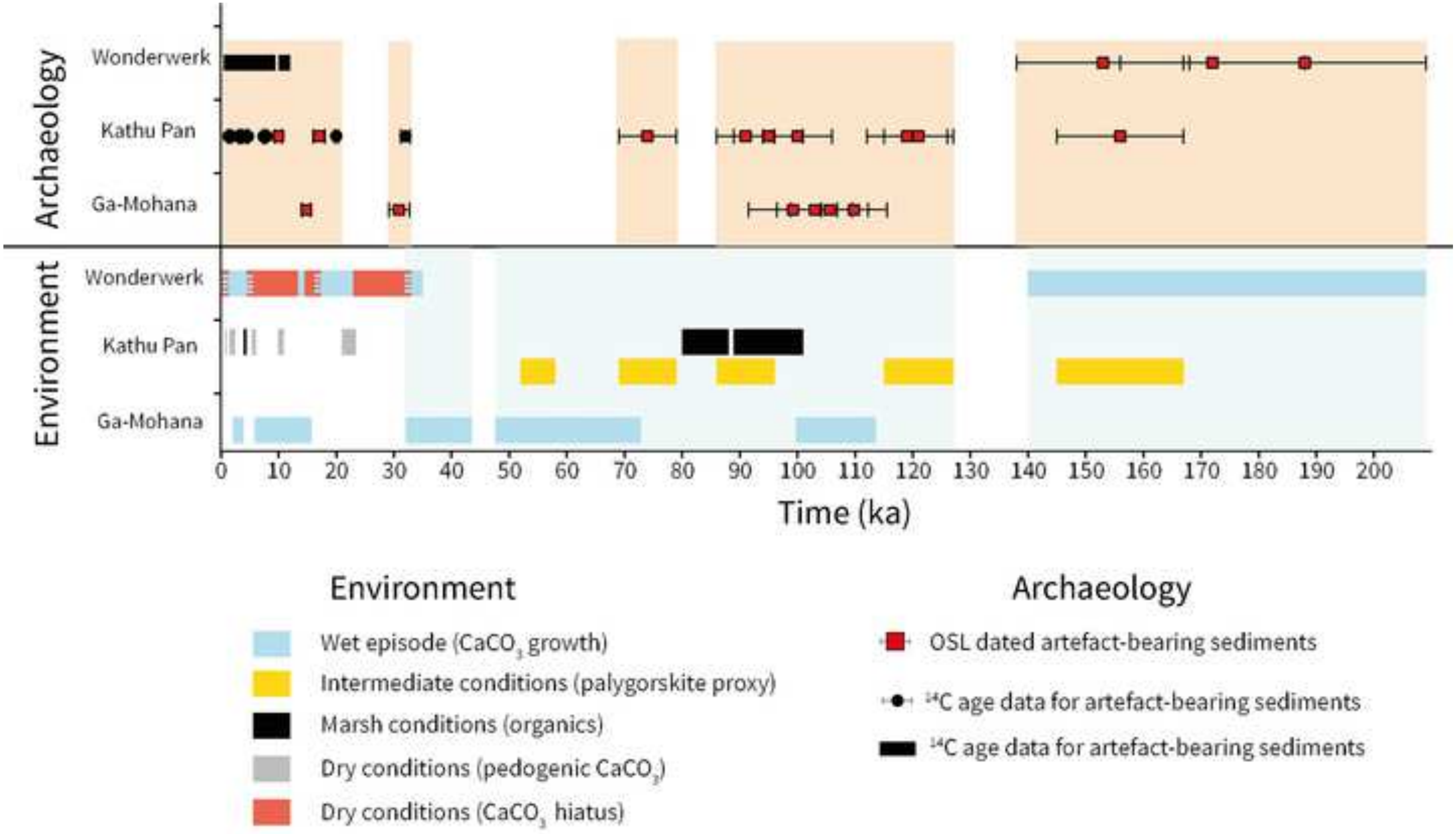

S1 Table

Click here to access/download
Supporting Information
S1_Table.pdf

S1 Figure

Click here to access/download
Supporting Information
S1_Fig.pdf

S2 Figure

Click here to access/download
Supporting Information
S2_Fig.pdf

S3 Figure

Click here to access/download
Supporting Information
S3_Fig.pdf

S4 Figure

Click here to access/download
Supporting Information
S4_Fig.pdf

S5 Figure

Click here to access/download
Supporting Information
S5_Fig.pdf

S6 Figure

Click here to access/download
Supporting Information
S6_Fig.pdf

S7 Figure

Click here to access/download
Supporting Information
S7_Fig.pdf

S8 Figure

Click here to access/download
Supporting Information
S8_Fig.pdf

S2 Table

Click here to access/download
Supporting Information
S2_Table.pdf

S3 Table

Click here to access/download
Supporting Information
S3_Table.pdf
